# Differential critical residues on the overlapped region of the non-structural protein-1 recognized by flavivirus and dengue virus cross-reactive monoclonal antibodies

**DOI:** 10.1038/s41598-022-26097-y

**Published:** 2022-12-13

**Authors:** Prasit Luangaram, Chamaiporn Tamdet, Chananya Saengwong, Tanapan Prommool, Romchat Kraivong, Napon Nilchan, Nuntaya Punyadee, Panisadee Avirutnan, Chatchawan Srisawat, Prida Malasit, Watchara Kasinrerk, Chunya Puttikhunt

**Affiliations:** 1 Molecular Biology of Dengue and Flaviviruses Research Team, Medical Molecular Biotechnology Research Group, National Center for Genetic Engineering and Biotechnology (BIOTEC), National Science and Technology Development Agency (NSTDA), Pathum Thani, Thailand; 2 Medical Biotechnology Research Unit, BIOTEC, NSTDA, Bangkok, Thailand; 3 Graduate Program, Department of Immunology, Faculty of Medicine, Siriraj Hospital, Mahidol University, Bangkok, Thailand; 4 Division of Dengue Hemorrhagic Fever Research, Faculty of Medicine Siriraj Hospital, Mahidol University, Bangkok, Thailand; 5 Siriraj Center of Research Excellence in Dengue and Emerging Pathogens, Faculty of Medicine Siriraj Hospital, Mahidol University, Bangkok, Thailand; 6 Department of Biochemistry, Faculty of Medicine Siriraj Hospital, Mahidol University, Bangkok, Thailand; 7 Biomedical Technology Research Center, BIOTEC, NSTDA, Chiang Mai, Thailand; 8 Division of Clinical Immunology, Department of Medical Technology, Faculty of Associated Medical Sciences, Chiang Mai University, Chiang Mai, Thailand

**Keywords:** Biochemistry, Biological techniques, Immunology, Molecular biology

## Abstract

The non-structural protein-1 (NS1) of dengue virus (DENV) contributes to several functions related to dengue disease pathogenesis as well as diagnostic applications. Antibodies against DENV NS1 can cross-react with other co-circulating flaviviruses, which may lead to incorrect diagnosis. Herein, five anti-DENV NS1 monoclonal antibodies (mAbs) were investigated. Four of them (1F11, 2E3, 1B2, and 4D2) cross-react with NS1 of all four DENV serotypes (pan-DENV mAbs), whereas the other (2E11) also reacts with NS1 of other flaviviruses (flavi-cross-reactive mAb). The binding epitopes recognized by these mAbs were found to overlap a region located on the disordered loop of the NS1 wing domain (amino acid residues 104 to 123). Fine epitope mapping employing phage display technology and alanine-substituted DENV2 NS1 mutants indicates the critical binding residues W115, K116, and K120 for the 2E11 mAb, which are conserved among flaviviruses. In contrast, the critical binding residues of four pan-DENV mAbs include both flavi-conserved residues (W115 to G119) and DENV-conserved flanking residues (K112, Y113, S114 and A121, K122). Our results highlight DENV-conserved residues in cross-reactive epitopes that distinguish pan-DENV antibodies from the flavi-cross-reactive antibody. These antibodies can be potentially applied to differential diagnosis of DENV from other flavivirus infections.

## Introduction

Dengue virus (DENV) is a mosquito-borne virus that causes dengue disease, which is a major public health problem in tropical and subtropical countries. Most DENV infections are asymptomatic; however, disease symptoms can range from an undifferentiated fever in mild dengue fever (DF) to life-threatening dengue hemorrhagic fever (DHF) that manifests vascular leakage, internal organ failure, and shock^1^. DENV comprises four serotypes (DENV1 − 4), belonging to the *Flavivirus* genus, family *Flaviviridae*, which includes Yellow Fever virus (YFV), Japanese Encephalitis virus (JEV), West Nile virus (WNV), and Zika virus (ZIKV). Antibodies against DENV can cross-react with other co-circulating flaviviruses and complicate diagnosis^2^. The pathogenesis of DENV infection is poorly defined and the imbalance of protective and pathogenic immune responses remains unclear.

The DENV genome contains three structural genes (C, PrM, and E) and seven non-structural genes (NS1, NS2a, NS2b, NS3, NS4a, NS4b and NS5). Non-structural protein 1 (NS1) plays a crucial role in DENV replication and is involved in the pathogenesis of DENV infection^3^. Immunization of mice with NS1 or passive transfer of anti-NS1 antibodies confers protection against DENV challenge^4,5^, highlighting the potential of NS1 as a vaccine candidate with low to no risk of antibody-dependent enhancement (ADE), which is typically associated with structural protein-based vaccines^5^. Since NS1 can cause both pathogenic and protective roles, therefore engagement of protective epitopes and/or elimination of pathogenic epitopes should be considered in dengue NS1 vaccine design. NS1 is expressed in various forms during DENV infection, i.e. intracellular monomeric and dimeric membrane-bound, and secreted hexameric NS1^6^. NS1 is a good biomarker for early dengue diagnosis since it can be detected in the blood circulation of DENV-infected patients, especially during the febrile phase^7,8^. Several DENV NS1 antigen kits (either ELSA or immunochromatographic test format) employing anti-NS1 antibodies have been commercialized worldwide.

Anti-NS1 antibodies bind to various B-cell epitopes on NS1, many of which have been identified from either immunized mice or DENV-infected patients^3^. The epitopes include DENV serotype-specific (DENV-SS) and serotype cross-reactive epitopes present among all four serotypes (pan-DENV). Pan-DENV epitopes have been identified on several NS1 regions, including the β-roll domain and connector subdomain (aa 21–35^9,10^), the wing domain (aa 110–125^10–13^ and aa 141–168^14^), and the β-ladder domain (aa 190–205^10,15^, aa 261–275^10^ and aa 276–305^10,16^). Owing to the 50–70% similarity of flavivirus NS1 sequences^17^, antibodies induced by DENV NS1 can cross-react with other flaviviruses (Flavi-cross-reactive). Therefore, differential B-cell epitope mapping of cross-reactive mAbs is crucial for assessing clonal identity, as well as for the development of diagnostic assays to distinguish DENV from other flaviviruses, especially in the endemic areas where multiple flaviviruses co-circulate.

We previously produced mouse anti-DENV NS1 mAbs, including five (2E11, 1F11, 2E3, 1B2 and 4D2) that recognize linear epitopes on the DENV NS1 protein^18,19^. Four of these mAbs (1F11, 2E3, 1B2, and 4D2) are pan-DENV mAbs that recognize only DENV NS1, whereas 2E11 is a flavi-cross-reactive mAb. The molecular basis for the differences among these mAbs in specificity for different flavivirus NS1 is unknown. In this study, the precise binding epitopes of these mAbs are mapped to an overlapping region of the disordered loop of the NS1 wing domain and critical residues that distinguish pan-DENV antibodies from the flavi-cross-reactive antibody 2E11 are identified by fine epitope mapping.

## Results

### Characteristics of anti-NS1 mAbs binding to DENV and other flavivirus NS1

**Figure 1:**
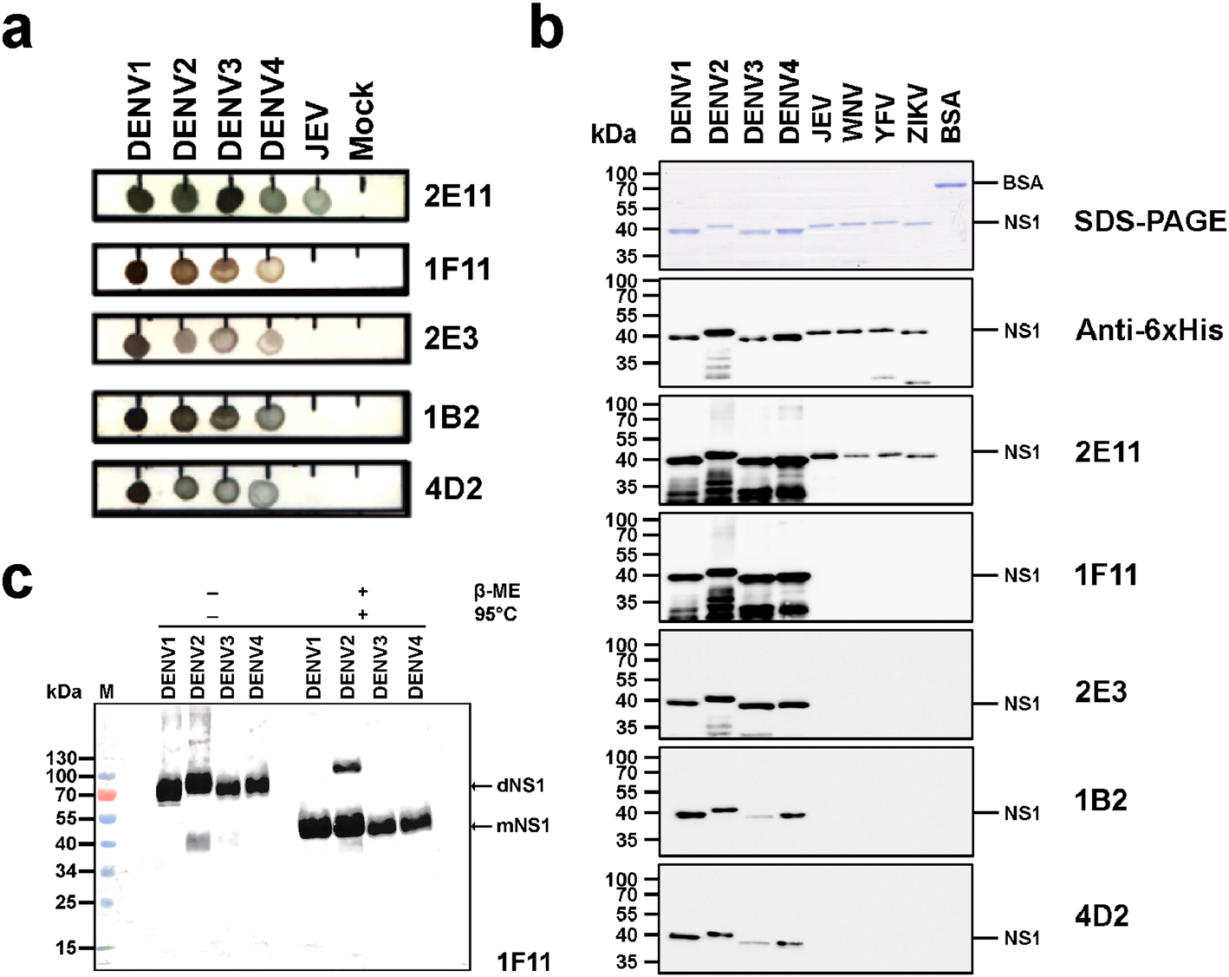
Characterization of anti-NS1 mAbs binding to DENV and flavivirus NS1. (**a**) Reactivity to soluble (secreted) NS1 of DENV1 − 4 and JEV by dot blot assay. Culture supernatants of C6/36 cells infected with DENV1, DENV2, DENV3, DENV4, JEV, or none (mock) were dotted onto a nitrocellulose membrane strip and reacted with indicated mAbs by dot blot assay. (**b**) Reactivity to NS1 proteins of DENV1 − 4 and other flaviviruses by western blot analysis. The purified rNS1 proteins of DENVs and other flaviviruses (1 µg each) expressed from *E. coli* were separated in SDS-PAGE and stained with Coomassie blue R250 (upper panel). Bovine serum albumin (BSA) was included as a negative control antigen. Proteins were blotted and reacted with anti-NS1 or anti-6His mAbs by western blot analysis. (**c**) Testing of conformation-dependent recognition by anti-NS1 antibodies. Purified rNS1 of DENV1 − 4 were pre-treated ( +) by β-mercaptoethanol (β-ME) and heat (95 °C) or no treatment (−) and analyzed by western blot with anti-NS1 mAbs (1F11). The antigen–antibody complexes were detected by anti-mouse IgG-HRP and visualized using chemiluminescent substrate. DENV1 to DENV4 (DENV serotype 1–4), JEV (Japanese encephalitis virus), WNV (West Nile virus), YFV (Yellow fever virus), ZIKV (Zika virus). Protein species corresponding to dimeric NS1 (dNS1; 90 kDa) and monomeric NS1 (mNS1; 45 kDa) are indicated. The molecular sizes (kDa) of standard proteins are shown on the left.

The binding specificity of five anti-NS1 mAbs was initially assessed by dot blot analysis. All five mAbs demonstrated cross-reactivity to secreted NS1 of all four DENV serotypes (DENV1 − 4), but only 2E11 showed reactivity to secreted NS1 of JEV (Fig. 1a), consistent with previous reports^18,19^. In addition, we utilized a panel of recombinant histidine-tagged NS1 proteins of DENV1 − 4 and other flaviviruses to test the cross-reactivity of mAbs to different flavivirus NS1 proteins. A western blot analysis showed that all five mAbs react with recombinant NS1 of all four DENV serotypes, albeit to various extents, and only 2E11 is reactive to recombinant NS1 of other flaviviruses including JEV, WNV, YFV and ZIKV (Fig. 1b). To test whether the reactivity of the 1F11 mAb is NS1 conformation-dependent, western blot was performed with DENV NS1 proteins pre-treated with reducing agent and heating prior to SDS-PAGE and untreated protein (non-reduced/no heat condition). NS1 protein was detected with the 1F11 mAb under both conditions (Fig. 1c). Another four mAbs, similar to 1F11, also gave the same pattern of reactivity (data not shown). The results indicate that they recognize linear epitopes in a manner independent of NS1 protein conformation. The kinetic binding parameters of the mAbs to four DENV NS1 recombinant proteins were determined by surface plasmon resonance technology. The mAbs exhibited strong binding affinity to DENV NS1 with dissociation constant (K_D_) in the nanomolar range (Table S1, Fig. S1a–c). The 2E3 did not bind NS1 on the sensor chip, similar to the 4G2 negative control (Fig. S1d–g). Thus binding parameters of 2E3 could not be determined.

### Anti-NS1 mAbs bind overlapping epitopes on the NS1 protein

We used a competitive binding ELISA to determine whether the anti-NS1 mAbs recognize epitopes distinct from one another, using the rationale that the binding of one mAb could compete with the binding of a second mAb if the epitopes overlap. A set of unlabeled competitor mAbs was used to block their binding epitopes on immobilized NS1 molecules. A set of FITC-labelled detecting mAbs comprising 2E11^F^, 1B2^F^, 4D2^F^, 1F11^F^ and 2E3^F^ was used to measure their binding to the blocked NS1. Binding competition was measured as the percent inhibition of FITC-labeled mAb signal. Self-blockings (competition with unlabeled mAb of the same type) showed greater than 70% inhibition of the FITC-labeled mAbs, which was used as a reference point for completely overlapping epitopes. Based on these competitive binding results, the anti-NS1 mAbs were classified into three groups (Table 1). 2E11 is classified as “Group A” due to its distinctly strong inhibition of all other detecting mAbs. Notably, the other four mAbs are not able to block the binding of 2E11^F^. This could be due to a pentameric IgM structure and a greater binding affinity of 2E11 to DENV2 NS1 than that of other four monomeric IgG mAbs (Table S1). 1F11 and 2E3 are classified as “Group B” as they strongly inhibit each other, and moderately inhibit the binding of 1B2^F^ and 4D2^F^ (30 − 60% inhibition). 1B2 and 4D2 are classified as “Group C” because they strongly inhibit each other as well as 1F11^F^ and 2E3^F^. These results suggest that binding epitopes of these five anti-NS1 mAbs are not exclusive of one another and occupy the same or overlapping regions of NS1.

**Table 1:** Analysis of distinct binding epitope group of anti-NS1 mAbs by competitive binding ELISA.

Blocking mAbs	Reactivity to NS1 of DENVs	Group Classification	% Inhibition of the FITC-labeled mAbs				
			2E11^F^	1F11^F^	2E3^F^	1B2^F^	4D2^F^
2E11	Flavi-cross reactive	A	**89.49**	96.21	95.36	94.70	86.15
1F11	DENV1−4	B	0.05	**87.66**	82.99	54.00	34.26
2E3	DENV1−4		1.80	67.53	**75.55**	51.53	30.84
1B2	DENV1–4	C	0.98	81.62	80.25	**84.23**	84.53
4D2	DENV1–4		9.42	83.22	85.12	88.67	**80.37**

Interpretation of binding epitope between each pair of antibodies (blocking and FITC-labeled mAbs); Completely overlapping epitope: > 70% inhibition, Partial overlapping epitope: 20–70% inhibition, Discrete or non-overlapping epitope: < 20% inhibition. Bold values indicate self-blocking.

### Anti-NS1 mAbs recognize highly conserved regions in the NS1 wing domain

**Figure 2:**
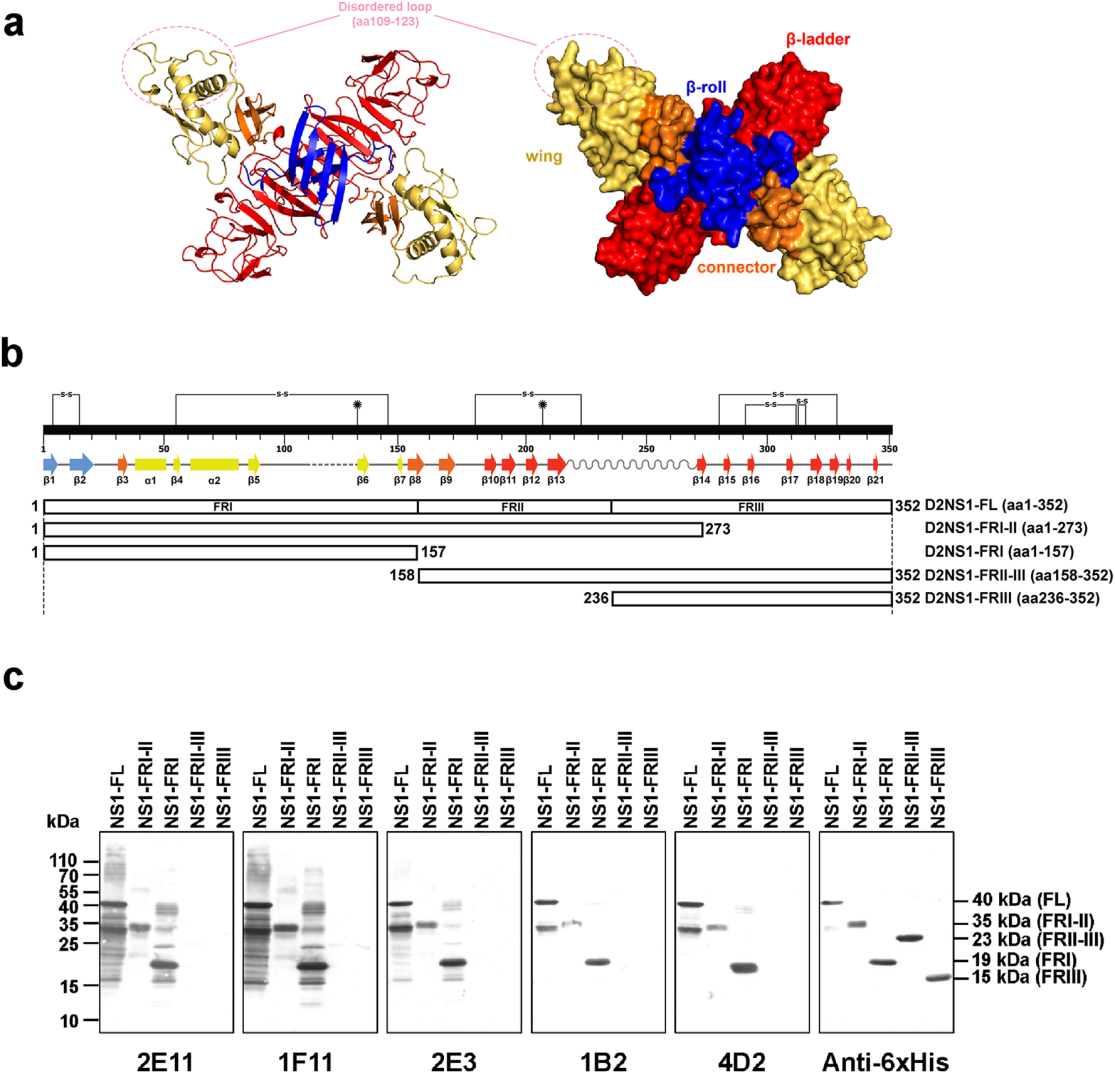
Mapping of NS1 fragments recognized by anti-NS1 antibodies. (**a**) Dimeric DENV2 NS1 structure. Each monomer contains the domains: β-roll (blue, aa 1 − 29), Wing (yellow, 38 − 151) and β-ladder (red, aa 181 − 352) connected by discontinuous subdomains (orange, aa 30 − 37 and aa 152 − 180). Secondary structure view (left) and three-dimensional structure view (right). The NS1 structure was modeled with SWISS-MODEL^34,35^. An input of full-length amino acid sequence of DENV2 NS1 were used in order to depict missing residues in the template crystal structure (PDBID: 4O6B). PyMol was used for structure visualization (The PyMOL Molecular Graphics System, Version 2.0 Schrödinger, LLC.). (**b**) The schematic diagram of recombinant truncated NS1 fragments: The full-length DEN2NS1 (NS1-FL) (aa 1 − 352) can be divided into three fragment domains (FRI, FRII, and FRIII). Four truncated DEN2NS1 recombinant proteins spanning these fragments were generated: NS1-FRI, (aa 1 − 157); NS1-FRI-II (aa 1 − 273); NS1-FRII-III (aa 158 − 352), and NS1-FRIII (aa 274 − 352). The DENV2 NS1 secondary structure elements and corresponding amino acid residues are shown above. (**c**) Reactivity of anti-NS1 mAbs to rNS1-FL and its truncated fragments. *E. coli* lysates expressing various rNS1 fragments were analyzed by western blot with five anti-NS1 mAbs indicated below each panel (2E11, 1F11, 2E3, 1B2, and 4D2). Equivalent amounts of rNS1 fragments were loaded in each lane as shown by western blot with anti-6His antibody. The molecular sizes in kDa of all fragments are shown on the right.

The NS1 dimeric structure contains three distinct domains (β-roll, Wing and β-ladder) on each monomer (Fig. 2a). To map the mAb binding regions, recombinant plasmids containing genes encoding for full-length DENV2 NS1 (rNS1-FL) or various truncated NS1 fragments (FRI, FRI-II, FRII-III and FRIII) encompassing three distinct domains as shown in Fig. 2b were generated. Expression of the recombinant proteins in *E. coli* was determined by western blot assay using anti-6His antibody (Fig. 2c, most right panel). All five anti-NS1 mAbs reacted with rNS1-FL (45 kDa), rNS1-FRI-II (35 kDa) and rNS1-FRI (19 kDa), but not FRII-III (23 kDa) and FRIII (15 kDa) (Fig. 2c). These results suggested that all mAbs bind to the FRI fragment, which includes the first 157 amino acid residues covering the β-roll and wing domains of NS1.

We further utilized a panel of 15-mer synthetic oligopeptides to narrow down the mAb binding epitopes on the FRI fragment. The 15-mer oligopeptides with five-residue overlap covering aa 1–173 (Table S2) were coated on an ELISA plate and reacted with four anti-NS1 mAbs – 2E11, 1F11, 1B2, and 2E3. The 4D2 was not included in this experiment owing to a limited amount of oligopeptides. All four tested mAbs showed a positive reaction with the peptide #32 (aa 109–123; TELKYSWKTWGKAKM), whereas only 1F11 showed a reaction with an additional peptide #8 (aa 104–118; LRPQPTELKYSWKTW). This result indicates that the binding epitopes of these four mAbs are located around aa 104–123 of NS1 with overlap aa 109–118.

### Epitope mapping by phage display peptide library of flavi-cross-reactive 2E11 and pan-DENV 1F11 mAbs identify critical binding residues within aa 112–120 of DENV NS1

**Figure 3:**
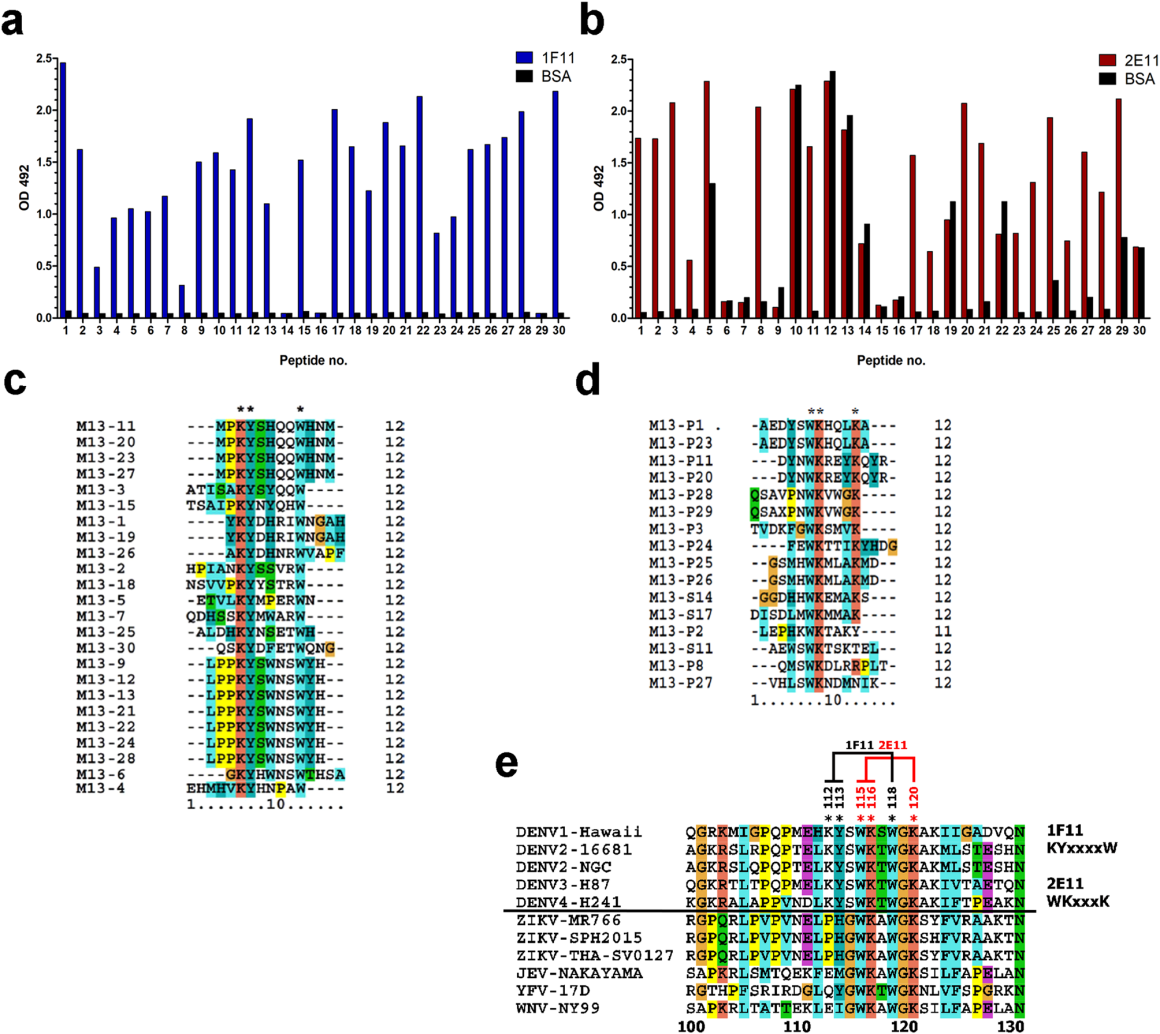
Mapping of anti-NS1 antibody binding epitopes by 12-mer phage surface display library. Selected phage clones obtained from phage peptide library biopanning with 1F11 (**a**) or 2E11 (**b**) were reacted with antibody-coated ELISA, followed by HRP-conjugated anti-M13 antibodies. BSA was used as a negative control protein. The 12-mer peptide sequences of twenty-four 1F11-immunopositive phage clones (**c**) and sixteen 2E11-immunopositive phages (**d**) were ascertained and aligned. Conserved residues among all phage clones of each antibody are marked by asterisks. (**e**) Alignment of NS1 aa sequences of DENV and flaviviruses from aa 99 to 129. The binding motifs of 1F11 (KYxxxxW) and 2E11 (WKxxxK) are indicated together with DENV-conserved residues matching the 1F11 motif (K112, Y113, and W118), and flavivirus-conserved residues W115, K116 and K120 matching the 2E11 motif.

To identify potential binding residues of the mAbs, we initially used a Ph.D.-12 phage library displaying 12-mer peptides with 2E11 (flavi-cross-reactive) and 1F11 as a representative of pan-DENV mAbs. Immuno-positive phage clones from biopanning with 1F11 (n = 26/30) and 2E11 (n = 18/30) were identified by monoclonal phage ELISA (Fig. 3a, b), and their DNA sequences were analyzed. The amino acid sequences of twenty-four 1F11-immunopositive phages revealed three repeated peptide sequences: MPKYSHQQWHNM (n = 4), YKYDHRIWNGAH (n = 2) and LPPKYSWNSWYH (n = 7), and 11 unique peptides (Fig. 3c). The consensus sequence of these peptides suggests a potential binding motif of 1F11 as **KY**xxxx**W**, which corresponds to aa 112 to 118 on DENV NS1 (Fig. 3e). Among sixteen 2E11-immuno-positive phages, four repeated peptide sequences: AEDYSWKHQLKA (n = 2), DYNWKREYKQYR (n = 2), QSAVPNWKVWGK (n = 2), GSMHWKMLAKMD (n = 2), and 8 unique peptides were apparent (Fig. 3d). The consensus sequence of these peptides indicated a potential binding motif of 2E11 as **WK**xxx**K**, which corresponded to aa 115 to 120 on DENV NS1 (Fig. 3e). According to the aa sequence alignment among flaviviruses, all three residues (W115, K116, and K120) of 2E11 binding motif, together with W118 and G119, are conserved among flaviviruses, whereas the first two residues (K112 and Y113) of the 1F11 binding motif are DENV-specific (Fig. 3e).

To assess the importance of these conserved residues on NS1 sequences for mAb binding, various oligopeptides covering aa 110–122 of DENV and/or other flavivirus NS1 were synthesized and used in peptide-coated ELISA (Fig. S2d). Positive binding reactivity was determined when the OD reading is above a cut-off value (twice OD reading to P-capsid, a negative control peptide). Both 1F11 and 2E11, as expected, showed the highest reactivity to the peptide P2-D2/3, covering wild-type sequences of DENV2 and 3 NS1 and slightly less reactivity with the peptide P1-D1 corresponding to the epitope in DENV1 NS1. The pan-DENV mAb 1F11 did not react with other flavivirus NS1 peptides, derived from JEV, WNV and YFV (P3-JE, P4-WN, and P5-YF, respectively) (Fig. S2a). In contrast, the flavi-cross-reactive mAb 2E11 reacted with P3-JE and P4-WN, but not P5-YF (Fig. S2b).

Regarding DENV2 NS1 variant oligopeptides with substituted residues (Fig. S2a), 1F11 showed markedly less binding to the peptide MT-1F11, which has alanine substitution of three conserved residues (_112_**KY**XXXX**W**_118_ to _112_**AA**XXXX**A**_118_**),** and the peptides with alanine-substitutions of individual conserved residues (D2-K112A, D2-Y113A, and D2-W118A). Likewise, a complete loss of 2E11 binding was observed for the peptide MT-2E11 with alanine substitution of all three conserved residues (_115_**WK**XXX**K**_120_ to _115_**AA**XXX**A**_120_), as well as the peptides with single substitutions (D2-W115A, D2-W116A, and D2-K120A) (Fig S2c). From these results, we infer that the conserved residues are essential for mAb binding (K112, Y113, and W118 for 1F11; W115, K116, and W120 for 2E11).

### Fine mapping of critical binding residues for other anti-NS1 mAbs

**Figure 4:**
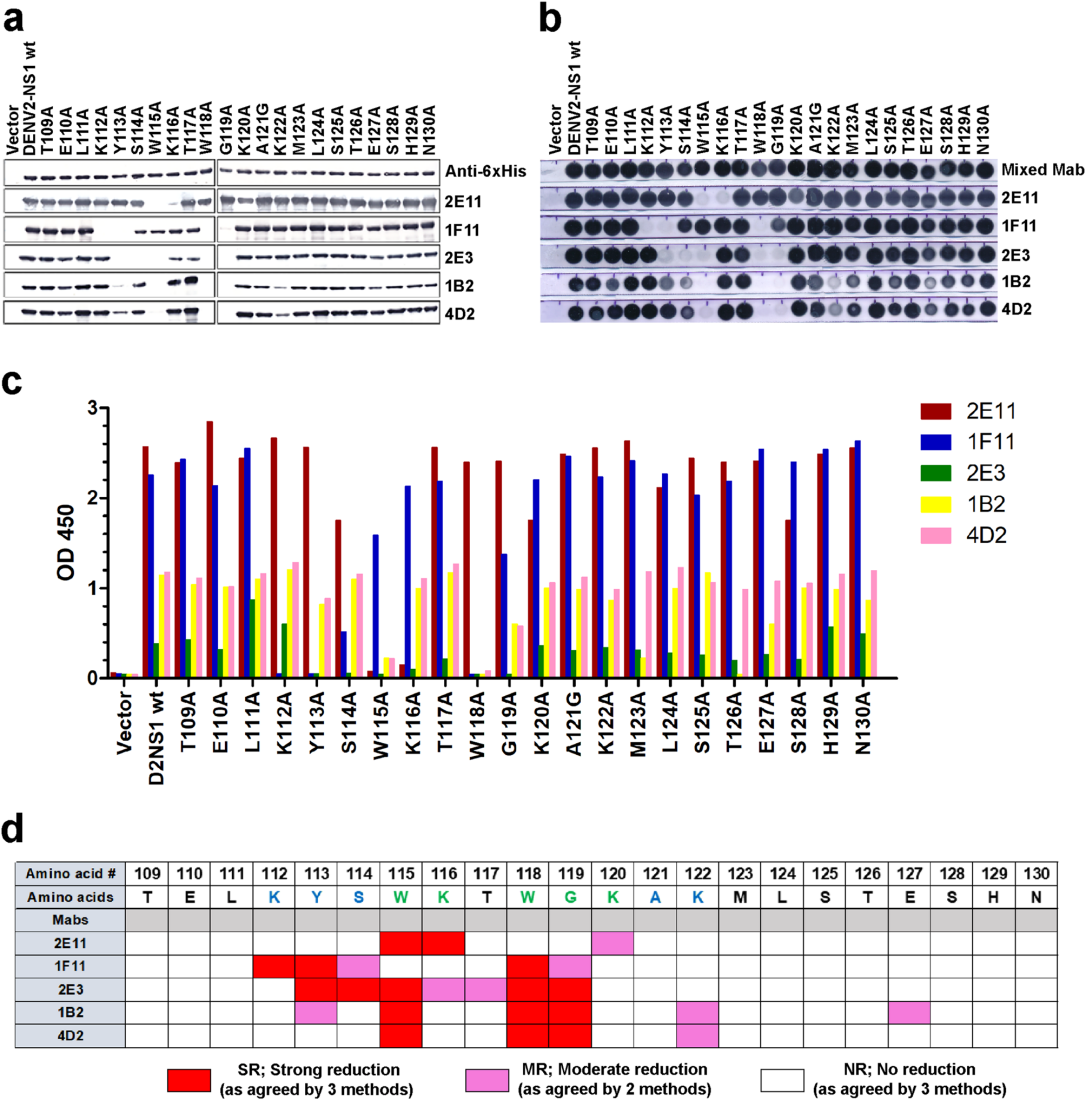
Reactivity of anti-NS1 antibodies to alanine-substituted rDENV2 NS1 mutant proteins. A set of rNS1-6His mutants in which residues 109−130 were substituted by alanine (or glycine for residue 121) were expressed in *E. coli*. The bacterial lysates of mutants were reacted with anti-NS1 antibodies by (**a**) western blot analysis or (**b**) dot blot assay. Equivalent amounts of rNS1 mutant proteins were applied, as shown by anti-6His antibody (a, upper row) or a mixture of 16 anti-NS1 mAbs that recognized different epitopes on NS1 protein (b, upper row). (**c**) NS1 captured ELISA. Alanine-substituted NS1 mutants (aa 109 − 130) were expressed from transfected imHC cells. Each rNS1 mutant from culture supernatant was captured with anti-6His mAb coated wells and reacted with anti-NS1 mAbs as indicated on the key (2E11, 1F11, 2E3, 1B2 and 4D2) and measured at OD_450_. Cultures from cells transfected with plasmid containing wild-type DENV2 NS1 (NS1-wt) or empty vector were used as a positive or negative control, respectively. (**d**) Assignment of critical binding residues from analysis of mutants. The degree of signal reduction in mutants was determined based on the relative binding index (RI) either by western/dot blot signal intensity or OD reading of NS1 mutant to NS1 wt. i.e. Strong reduction (SR; RI was less than 0.3), moderate reduction (MR; RI was 0.3–0.6) or negligible reduction (NR; RI was greater than 0.6). Substituted residues assigned as SR (Red), MR (Pink), or NR (no color) were congruent by at least 2 assays. NS1 residues conserved among DENV1-4 (blue) or flaviviruses (green) are indicated.

**Figure 5:**
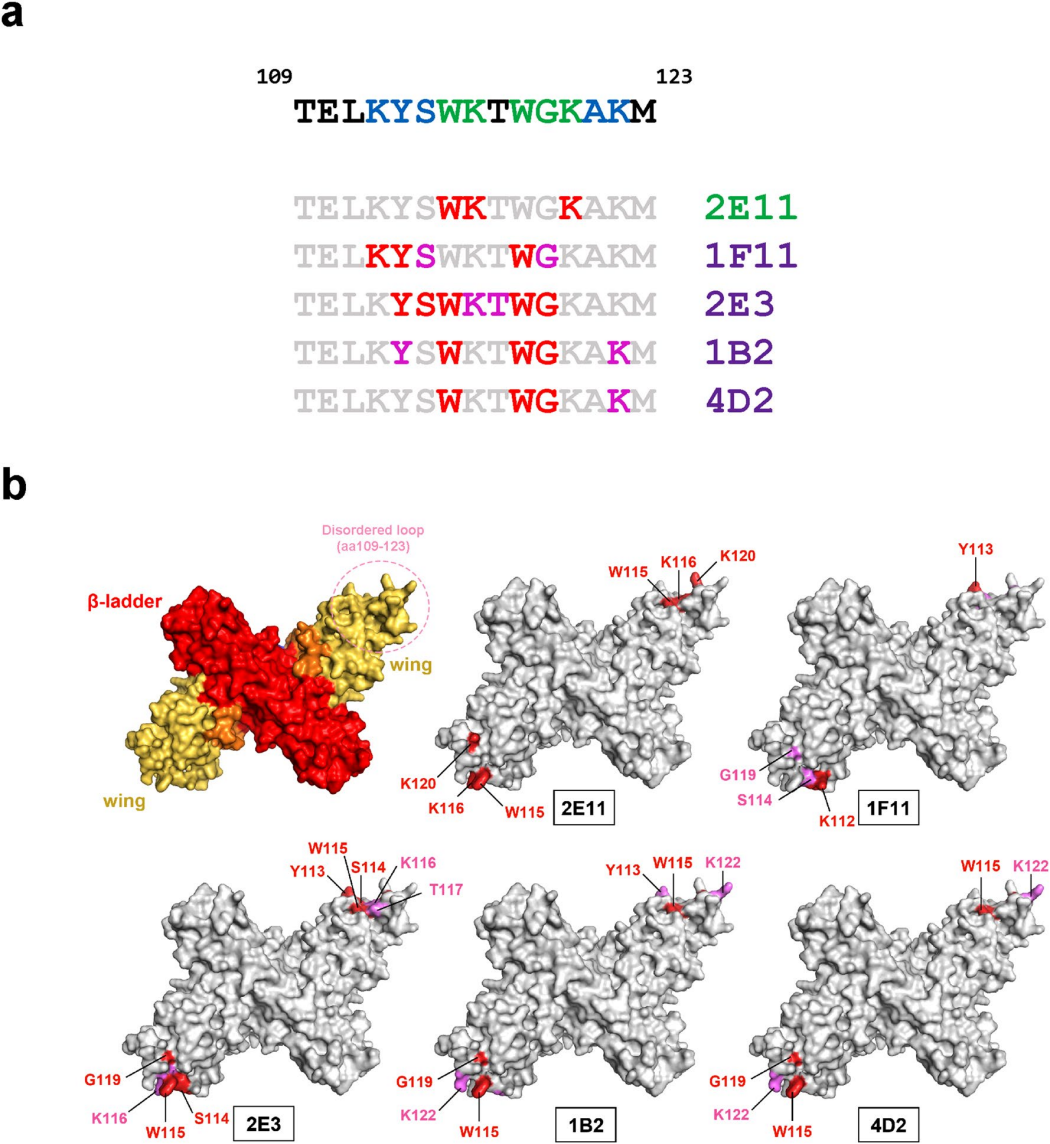
Summary of critical binding residues on DENV NS1 by anti-NS1 mAbs. (**a**) Amino acid sequence of DENV2 NS1 between position 109–123 containing residues conserved among DENV1 − 4 (blue) and flaviviruses (green). The critical binding residues of five anti-NS1 antibodies are highlighted in red (strong) and pink (moderate). (**b**) Critical binding residues of each mAbs were depicted on dimeric NS1 structures in PyMol; strong (red) and moderate residues (pink) are indicated.

We further identified the key binding residues of the other three anti-NS1 mAbs using full-length alanine-substituted NS1 proteins. 1F11 and 2E11 were used to verify the reactivities of these recombinant proteins. The recombinant NS1 (rNS1) mutants, of which aa 109–130 were individually mutated to alanine or glycine, were expressed in two systems, i.e. *E. coli* lysate, and culture supernatant of transfected immortalized hepatocyte-like cells (imHC)^20^. The binding reactivities of the anti-NS1 mAbs to rNS1 mutants derived from bacterial cell lysate were analyzed by western blot (Fig. 4a) and dot blot assays (Fig. 4b), whereas the binding of antibody to secreted rNS1 mutants produced from mammalian cells was analyzed by anti-6His capture ELISA (Fig. 4c). The relative binding index (RI) was calculated for each mAb binding to rNS1 mutants (either western/dot blot signal intensity or ELISA OD reading) with respect to an equivalent amount of wild-type rNS1 (Fig. S3–S5). The degree of reduction of each mutant NS1 proteins were graded according to RI: strong reduction of binding (SR, red color) with RI < 0.3, moderate reduction (MR, pink color) with RI = 0.3 − 0.6 and no reduction (NR, no color) with RI > 0.6, as shown in Table S3. As confirmed by at least two assays, the reduction levels of each mAb binding to the mutants are summarized in Fig. 4d. The rNS1 mutants showing strong (red) and moderate (pink) reduction of mAb binding have substitutions of critical mAb binding residues. In comparison to the results from phage display (Fig. 3) and peptide ELISA (Fig. S2), the critical residues defined by these full-length rNS1 mutants were identical for 2E11 (W115, K116, and K120) and 1F11 (K112, Y113, and W118), whereas additional residues S114 and G119 were also found for 1F11 binding (Fig. 4d). We then analyzed critical binding residues for 2E3, 1B2, and 4D2 by using full-length rNS1 mutants. Each mAb binds to different critical residues between 113 and 122, which includes DENV and flavi-conserved residues (Fig. 4d). Only 1B2, critical residue is also extended to 127. In summary, the footprints of critical binding residues of these five mAbs are identified and highlighted in Fig. 5a. They are conserved among DENV and flavivirus NS1 within the overlapped epitopes (aa 109–123) reside on the disordered loop of the dimeric NS1 structure as shown in Fig. 5b. While the binding of 2E11 is focused on the flavi-conserved region (W115-W120), flanking DENV-conserved residues are important for binding of the four pan-DENV mAbs (K112, Y113, S114, A121 and K122).

**Figure 6:**
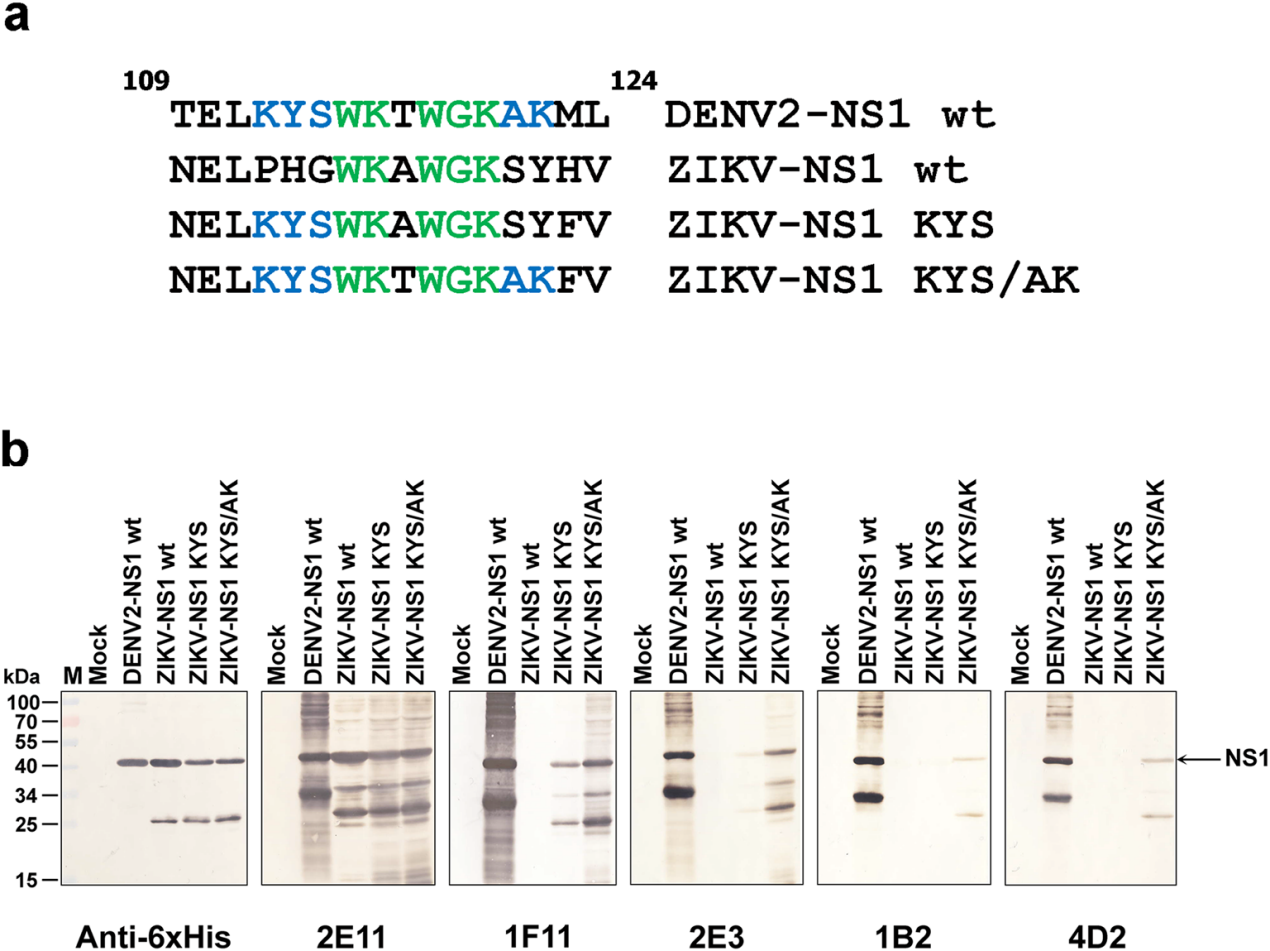
Reactivity of anti-NS1 antibodies to recombinant Zika NS1 proteins. (**a**) Comparison of NS1 aa sequences 109–124 of DENV2-NS1, ZIKV-NS1 wild type (wt.) and two mutants (ZIKV-NS1 KYS and ZIKV-NS1 KYS/AK). Flavi-conserved residues (green) and DENV-conserved residues (blue) are highlighted. (**b**) The rNS1 proteins in *E. coli* lysates were reacted with anti-NS1 antibodies as indicated below the panels by western blot analysis and visualized by DAB chromogenic substrate. Anti-6His antibody was used to confirm the expression of all rNS1 proteins.

To confirm the significance of DENV-conserved residues for binding of four pan-DENV mAbs, we created two ZIKV NS1 mutants with substitutions of these residues (Fig. 6a). The ZIKV NS1-KYS mutant contains DENV-conserved residues (_112_KYS_114_), while the other, ZIKV NS1-KYS/AK mutant carries DENV residues from K112 to K122. Expressions of rNS1 mutants, as well as wild-type (wt) DENV2 and ZIKV NS1 were confirmed by western blot assay with anti-6His mAb (Fig. 6b). Pan-DENV mAbs reacted with DENV-NS1 wt, but not ZIKV-NS1 wt. However, the ZIKV NS1-KYS/AK mutant can be detected by four pan-DENV mAbs with different extents, of which 1F11 showed the strongest reactivity. Whereas, the ZIKV NS1-KYS mutant was only reacted by 1F11, with less extent than the ZIKV NS1-KYS/AK mutant, but not by other mAbs. In contrast to the pan-DENV mAbs, the flavi-cross-reactive mAb 2E11 can bind similarly to all NS1 variants. These results confirm the importance of DENV-conserved residues for binding of four pan-DENV mAbs.

Due to the genotype concerns, single representative strains belonging to different genotypes of DENV serotype 1–4 were selected for analysis^21^. The NS1 amino acid sequences (aa 109–124) of these DENV strains as well as that of other flaviviruses were aligned as shown in Fig. S6. The alignment indicated that KYS/AK are conserved among all DENV genotypes, but not other flaviviruses. Whereas WKxWGK (115–120) are conserved for all flaviviruses including DENV. The result suggests that these pan-DENV mAbs are highly specific to DENV and can be applied in diagnosis purposes, especially in differential diagnosis of DENV from other flaviviruses.

### Anti-NS1 mAbs do not cross-react with host LYRIC epitope

Antibodies against NS1 can cross-react with host proteins (auto-antibodies), which may contribute to the pathogenesis of DHF/DSS. The human endothelial LYRIC protein is a known target of auto-antibodies^11^, as it contains a consensus anti-NS1 mAb binding motif KxWG corresponding to aa 116–119 of DENV NS1, where is the region mapped by our five anti-NS1 mAbs. Therefore, we tested if our mAbs exhibit cross-reactivity to LYRIC protein. The anti-NS1 mAbs were incubated with human umbilical vein endothelial cells or HUVECs (A gift from Dr Panisadee Avirutnan) which express LYRIC epitope on the cell surface. PECAM-1 and anti-E (4G2) antibodies were used as positive and negative controls, respectively. Strong fluorescent signal on the cell surface was found by PECAM-1 antibody, but no or fainted signal was observed by five anti-NS1 mAbs and 4G2 (Fig S7a). Fluorescent intensity of each antibody was quantified and normalized to nuclear staining signals in the same captured areas. The result shows no significant difference in log10-transformed means of normalized fluorescent intensity among all anti-NS1 mAbs and 4G2 (one-way ANOVA’s p-value = 0.46) indicating anti-NS1 mAbs do not significantly recognize LYRIC epitope on the cell surface (Fig S7b).

## Discussion

In this study, we comprehensively mapped binding epitopes of five anti-NS1 mAbs. Four of which – 1F11, 2E3, 1B2, and 4D2 – are specifically reactive to DENV, whereas the other mAb, 2E11, exhibits a broader reactivity towards NS1 of flaviviruses including JEV, WNV, YFV, and ZIKV (flavi-cross-reactive). Epitope mapping by competitive binding ELISA and direct binding to truncated NS1 fragments and NS1 oligopeptides showed that these mAbs recognize epitopes with putative binding residues mapping to the overlapping NS1 region aa 109–123, which is located in the disordered loop region of the wing domain^22^. The core flavi-conserved residues located in the NS1 region aa 115–120 are minimally required for the binding of flavi-cross-reactive mAbs, whereas the flanking DENV-conserved residues (K112, Y113, S114, A121, and K122) are essential for the binding of pan-DENV mAbs.

Although the key binding residues could be conclusively identified for each mAb, the reasons for the differences in mAb binding behavior among NS1 proteins from different virus species are unclear, suggesting that the recognition of epitopes by mAb can be affected by the NS1 protein context. In experiments where the DENV NS1 linear epitope (aa112–122) was expressed on the ZIKV NS1 backbone in the ZIKV-NS1 KYS/AK mutant, the reactivity of pan-DENV mAbs was demonstrated suggesting the importance of DENV-conserved residues for these mAbs. However, the reactivities of these mAbs were markedly lower compared with the DENV2 NS1 wt. (Fig. 6b), which might be explained by structural differences in the NS1 wing domain between DENV2 and ZIKV. A comparison of high-resolution NS1 wing domain structures indicated extensive positive charge and disordered distal tip for the DENV2 wing domain, whereas that of ZIKV shows a remarkably negative charge profile and a structured distal tip^23^.

B-cell epitopes located in the disordered loop of the NS1 wing domain have been shown to elicit anti-NS1 mAbs in mouse vaccination and natural human infection^12,24,25^. Moreover, passive immunization of these epitopes confers protection against DENV challenge in mice^13,22,25,26^. A potential drawback of using such epitopes as immunogens is mimicry to host protein. Notably, the NS1 epitope contains the KxWG motif (NS1 region aa 116 − 119), which is also present in the host LYRIC protein expressed on human endothelial cells^11^. In addition, the ELK/KEL-type motif present on human blood-clotting proteins (fibrinogen), platelets and endothelial cells, resembles NS1 at aa 110 − 112^27^. This epitope mimicry to host proteins can generate autoantibodies and may induce pathological effects on human platelets and endothelial cells, accounting for thrombocytopenia and vascular leakage during dengue disease^11,27,28^. Autoreactivity against LYRIC protein can be avoided using immunogens without the mimicry epitope; for example, passive immunization of 33D2 mAb specific to the modified NS1 aa 112 − 122 peptide (lacking LYRIC epitope) protects mice from hemorrhage and lethal DENV challenges^13^. In this study, our five mAbs do not bind to the LYRIC motif (KxWG), suggesting potential protective efficacy of these mAbs, although further in vivo investigation is still required.

The anti-NS1 mAbs investigated in this study have been widely used to identify DENV infection in our laboratory. The flavi-cross-reactive mAb 2E11 is used as a capture antibody for the DENV serotyping-NS1-ELISA in tandem with a set of serotype-specific anti-NS1 mAbs^19,29^. The pan-DENV mAb 1F11 is utilized in the ‘Cygnus’ NS1-based smartphone multiplex microcapillary device for rapid DENV serotyping^30^. Both assays can differentiate DENV serotypes in clinical specimens during the fever stage and are simple alternatives to conventional methods such as RT-PCR or virus isolation for identifying DENV serotypes.

In conclusion, overlapping B-cell epitopes of five anti-NS1 mAbs specific to the linear epitopes spanning over the disordered loop of NS1 wing domain were identified. Core residues 115 − 120 define the minimal element recognized by flavi-cross reactive mAbs whereas the flanking residues are essential for DENV specificity. The knowledge of these critical residues that discriminate pan-DENV from flavi-cross reactive mAbs can be applied to understand the role of anti-NS1 antibodies in flavivirus disease and develop better diagnostic tools.

## Materials & methods

### Viruses

Four serotypes of DENV; DENV1 (strain Hawaii), DENV2 (strain 16,681), DENV3 (strain H87), and DENV4 (strain H241), as well as JEV (strain Nakayama) were propagated in mosquito C6/36 cells (ATCC, CRL1660). The cells were cultured in Leibovitz’s L-15 medium supplemented with 10% fetal bovine serum (FBS; Hyclone, USA), 100 U/ml penicillin G and 100 μg/ml streptomycin (Thermo Fisher Scientific, USA) in a 28 °C incubator.

### Purification of secreted DENV NS1 protein

Cell culture supernatant of DENV infected Vero cells under serum-free condition was collected 5 − 7 days post-infection and centrifuged at 15,000×*g* for 1 h, followed with 203,000×*g* for 4 h. The supernatant was subjected to immuno-affinity chromatography using anti-NS1 antibody (2G6) coupled to Sepharose 4B beads^31^. Bound NS1 was eluted from beads with 20 mM diethylamine (DEA) pH 11.6 at a flow rate of 0.5 ml/min. The purity of eluted NS1 was assessed by SDS–polyacrylamide gel electrophoresis (PAGE). The protein concentration was determined by Qubit® protein assay (Thermo Fisher Scientific). Purified NS1 was stored in PBS (pH 7.4) and kept at − 80 °C until use.

### Antibodies

Anti-NS1 mAbs used in this study were 2E11 (or clone NS1-1F, IgM), 1F11 (or clone NS1-3F, IgG2a), 2E3 (or clone NS1-4F, IgG1)^18^, 1B2 (IgG1) and 4D2 (IgG1)^19^. Hybridoma cells producing these mAbs were cultured in either RPMI 1640 (Cytiva) supplemented with 10% FBS, 100 U/ml penicillin G and 100 μg/ml streptomycin or serum-free ISF-1 (PAN-Biotech, Germany), the latter of which is used for antibody purification. Cell supernatant containing mAbs was collected and kept at 4 °C until use. For antibody purification, cell supernatant was subjected to Hitrap protein G HP (for IgG isotypes) or IgM HP purification column (for IgM isotype) (Cytiva) as described in the manufacturer’s protocol. 1A9 mAb, which reacts with DENV capsid^32^, was used as an NS1-irrelevant antibody. Anti-6His antibody (HIS.H8; Thermo Fisher Scientific) was used to detect NS1-6H fusion proteins in all immunoassays.

### Competitive binding ELISA

Five anti-NS1 mAbs at various concentrations (1, 2, 4 μg/100 μl) or none (as a control), which were used as blocking mAbs, were added into ELISA wells, pre-coated with 125 ng purified DENV2 NS1, for 1 h at 37 °C Without disturbing the samples, 6 μl of 10 μg/ml (or 60 ng) anti-NS1 mAbs conjugated with fluorescein isothiocyanate (FITC), as a detection antibody (mAbs^F^), were directly added and binding reactions were incubated for another hour. After 3 times washing with PBS, rabbit anti-FITC-HRP (Sigma-Aldrich, USA) at a dilution of 1:2000 was added and reactions were incubated for 1 h. The ortho-phenylene diamine (OPD) substrate/H_2_O_2_ was added to develop color and the reaction was stopped by 4 N H_2_SO_4._ The OD reading at 492 nm was measured by ELISA plate reader. The percent blocking (% blocking) was calculated as:

$$ \% {\text{ blocking of mAbs }} = \, \left( {{\text{OD}}_{{{492}}} {\text{of the control well }}{-}{\text{ OD}}_{{{492}}} {\text{of the test well}}} \right) \, /{\text{ OD}}_{{{492}}} {\text{of the control well x 1}}00. $$

The OD_492_ of the control well was measured from the reactive well without blocking antibody, and the OD_492_ of the test well was from the well that was blocked by the highest concentration of blocking antibody. The degree of inhibition was graded according to % blocking (> 70%, strong; 25–70%, moderate; < 25%, weak or no inhibition). The binding of the mAbs^F^ with its non-labeled mAbs (self-blocking) was used as a positive control.

### Immunoblot analysis

NS1 proteins were separated by SDS-PAGE and transferred onto nitrocellulose membrane by electroblotting for western immunoblot assay. For dot blot assay, purified NS1 or bacterial lysates containing NS1 protein were dotted onto nitrocellulose membrane at the desired concentration. Membranes from both assays were blocked with 5% skim milk in PBS pH 7.4 for 1 h at room temperature. After 3 times washing with PBS, the blocked membranes were incubated with anti-NS1 mAbs for 1 h at 37 °C, followed by rabbit anti-mouse immunoglobulins conjugated with HRP (Agilent, USA) for another 1 h in the dark. To visualize the protein, the membranes were incubated with chromogenic substrate solution (Diaminobenzidine; DAB and H_2_O_2_) for 5 min. When greater sensitivity was required, the membranes were incubated with chemiluminescent substrate solution (Thermo Fisher Scientific) and exposed to X-ray film in the dark.

### Generation of recombinant NS1 proteins (rNS1)

Genes encoding full-length NS1 proteins of DENV1-4, ZIKV, WNV, YFV and JEV fused to 6His were cloned into the pET-21a expression vector (Novagen). Transformed *E. coli* (strain B834) containing these plasmids were grown and induced by 0.3 mM Isopropyl β-D-1-thiogalactopyranoside (IPTG) at 37 °C for 3 h to produce insoluble rNS1 proteins. To prepare purified rNS1 proteins, bacterial cells were harvested and lysed by sonication. The cell pellet was solubilized with 6 M guanidine denaturing buffer before loading into immobilized metal affinity chromatography (Talon; Takara Bio, USA), eluted by 8 M urea elution buffer (pH 4.0) and dialyzed in dialysis buffer for protein refolding. Expressions of rNS1 proteins were verified by SDS-PAGE and western blot analysis.

To generate recombinant truncated DENV2 NS1 fragments, genes encoding DENV2NS1-FL (full-length NS1; aa 1–352), DENV2NS1-FRI (aa 1–157), DENV2NS1-FRI-II (aa 1–235), DENV2NS1-FRII-III (aa 158–352), and DENV2NS1-FRIII (aa 236–352) were cloned into the pET-21a expression vector. Transformed *E. coli* (strain B834) containing these plasmids were induced by IPTG to produce recombinant truncated NS1 protein as described above. Protein expressions were verified by western blot analysis using anti-6His antibody.

### Construction of alanine-substituted NS1 mutants by site-directed mutagenesis

Genes encoding DENV2NS1 mutants where amino acids at the position 109 to 130 were individually substituted to alanine (A) or glycine (G) by PCR mutagenesis. Briefly, mutant NS1 genes were amplified from pET28a containing cloned DENV2NS1 DNA with various primer pairs that carried desired point mutations (Table S4) by Platinum™ Pfx DNA polymerase (Thermo Fisher Scientific). The PCR mutagenesis conditions were: pre-denaturation at 94 °C for 5 min, 35 cycles of denaturation at 94 °C for 15 s, annealing at 55 °C for 30 s, and elongation at 68 °C for 6.4 min, and final extension at 68 °C for 5 min. PCR products were treated with 10 U DpnI restriction enzyme (NEB, USA) for 2 h at 37 °C, followed by purification with QIAquick PCR Purification Kit (QIAGEN, Germany), and transformation into *E. coli* (strain DH5α). Bacterial clones containing mutant plasmids were selected and DNA sequences were verified by Sanger dideoxy sequencing (Macrogen Inc., Korea). For protein expression, the plasmids containing these mutant NS1 genes were transformed into *E. coli* (strain B834) prior to induction with IPTG. For testing the effects of alanine substitution on mAb binding, the immunoreactive signal to mutant NS1 was compared with that of an equivalent amount of wild-type DENV2 NS1. The loading of bacterial lysates produced from each transformant cell line which was adjusted to ensure equivalent levels of recombinant protein were quantified in each sample by Coomassie blue staining, verified by detection with either anti-6His antibody (Thermo Fisher Scientific) for western blot assay or by a mixture of our 16 anti-NS1 mAbs for dot blot assay.

For testing of soluble NS1 representing NS1 produced in infected mammalian cells and secreted into culture medium, genes for expression of alanine-substituted NS1 were generated and cloned into a mammalian expression vector. Briefly, pCAGGS-E28-NS1D2-(Gly4Ser)_2_-6His vector, which contains the last 28 amino acids of E protein (a natural signal sequence), the DENV2 NS1 gene tagged with a glycine-serine linker and six-histidine at the C-terminus, was used as a template for alanine substitution at amino acid residues 109 − 130 (aa 109 − 130). Site-directed mutagenesis was performed with specific primers for each mutant as described above. For protein expression, the plasmids encoding mutant NS1 genes were transfected into imHC cells (immortalized hepatocyte-like cell)^20^ with PEI transfection reagent (Sigma-Aldrich). Transfected cells were incubated for 3 days and cell supernatants containing soluble mutant NS1 proteins were collected and applied onto sandwich ELISA. Mouse anti-6His antibody was used as a capture antibody and chimeric anti-NS1 mAbs, in which the constant region of the original mouse antibody was replaced with that of human IgG1, were used as detecting antibodies. Rabbit anti-human IgG conjugated with HRP (Southern Biotech, USA) was used as a secondary Ab at a dilution of 1:6000, and the immunoreactive signal was obtained using TMB substrate and 2 N H_2_SO_4_. The signal was read at OD 450/620. The relative OD between mutant and wild-type NS1 protein was used for determining the effect of substituted residues on mAb binding.

### Production of recombinant ZIKV NS1 proteins

The NS1-encoding gene of ZIKV strain SV0127-14 (Accession #KU681081.3) was cloned into the pET28a expression vector to generate wild-type (wt.) ZIKV NS1. This plasmid was also used as a template for site-directed mutagenesis with specific primers (Table S5) to generate ZIKV NS1 KYS and ZIKV NS1 KYS/AK mutants, which carry substitutions of DENV2 KYS (aa 112–114) and KYS/AK (aa 112–122), respectively. After verification of mutations by DNA sequence analysis (Macrogen Inc.), plasmids were transformed into *E. coli* strain B834 for protein expression. Bacterial cell lysates of DENV2 NS1 wt, ZIKV NS1 wt and two ZIKV NS1 mutants were analyzed by western blot analysis with either anti-NS1 mAbs or anti-6His antibody as a positive control.

### NS1 peptide-ELISA

Two sets of oligopeptides covering different, but overlapping NS1 regions were synthesized (Mimotopes, Australia and Peptide 2 Inc., USA). The first set includes 15-mer oligopeptides covering aa 1 − 173 of DENV2 NS1 (Table S2). The other set includes 13-mer oligopeptides covering aa 110 − 122 of DENV NS1 mutants or other flavivirus (Fig S2d). ELISA wells were coated with oligopeptides (1 µg) at 4 °C overnight. After blocking with 3% BSA, anti-NS1 antibodies were added and incubated for 1 h at 37 °C. The peptide-antibody complex was detected with either rabbit anti-mouse immunoglobulin-HRP or goat anti-mouse IgM-HRP antibody (DAKO; Agilent) at a dilution of 1:1000. The plate was further incubated for 1 h at 37 °C, followed by the addition of OPD substrate/H_2_O_2_, and 4 N H_2_SO_4._ The color reaction was measured at OD 492 nm by ELISA plate reader. BSA or DENV2 capsid peptide was used as a negative control antigen or peptide. Two mAbs against DENV envelope (4G2) and capsid (1A9) proteins^32^ were used as negative control antibodies.

### Biopanning of phage-displayed peptides to anti-NS1 antibodies

Fine mapping of NS1 protein was performed by screening a phage display library comprising phage clones expressing 12-mer peptides on their surfaces (Ph.D.-12 phage display peptide library, NEB). The phage clones were biopanned with anti-NS1 mAbs (1F11 and 2E11). For 1F11, the antibodies (10 nM or 300 ng) were mixed with 10^9^ pfu/ml of phage library in a final volume of 200 µl and incubated at 37 °C for 20 min. Protein-G beads (GE Healthcare, Bio-Science, USA) were added to capture antibodies and further incubated for another 15 min, following by washing ten times with TBS buffer (50 mM Tris–HCl, 150 mM NaCl) containing 0.1% Tween-20 (TBST). Antibody-phage complexes were eluted and neutralized with 0.2 M glycine–HCl, pH 2.2) and 1 M Tris–HCl (pH 9.1), respectively. Eluted phages were incubated with *E. coli* ER2738 at the early log phase (OD_600_ = 0.01–0.05) for 4.5 h at 37 °C on a shaker incubator. After being centrifuged at 12,000×*g* for 10 min at 4 °C, supernatant containing phages was collected, precipitated with 20% PEG/2.5 M NaCl at 4 °C overnight, and centrifuged at 12,000×*g* for 10 min at 4 °C. The phage pellet was re-solubilized with 1xTBS buffer. The phages were titrated on LB agar containing IPTG and X-gal and isolated blue plaques were selected to calculate the phage titer. Another three rounds of biopanning were performed in the same manner. For 2E11, the antibodies were directly coated onto a 96-well plate and blocked with blocking buffer (0.1 M NaHCO_3_, pH8.6 and 5 mg/ml BSA). Phage clones were then added into the wells to selectively bind with 2E11 antibody. The bound phages were eluted, amplified, and characterized as described above.

### Selection of positive phage clones by ELISA

In the third round of biopanning, blue plaques from the LB plates were selected and individually inoculated with *E. coli* ER2738 to amplify phages. Binding reactivities of the peptides on the selected phages against either 1F11 or 2E11 were tested. Briefly, 25 μg/ml of either anti-NS1 antibodies (1F11 or 2E11) or BSA was coated onto a 96-well plate at 4 °C overnight and blocked with 2% BSA for 1 h. Single or pooled phage clones (3 phage clones per pool) in bacterial culture supernatant were added into the wells and further incubated for 1.5 h at room temperature. After washing, the antibody-bound phage clones were detected with anti-M13 antibody conjugated HRP (GE healthcare) diluted 1:5000 for 1 h at room temperature. The complexes were detected by OPD substrate/H_2_O_2_ and stopped with 4 N H_2_SO_4._ The color reaction was measured at 492 nm by ELISA plate reader. The phage supernatant that gave the OD reading to their target antibodies over the control BSA were identified as positive clones. The phage DNA was extracted for Sanger dideoxy DNA sequencing (Macrogen Inc.). Sequences were analyzed using ClustalX2 software^33^.

### Immunofluorescence surface staining

Human umbilical vein endothelial cells (HUVECs) were grown in 8-well chamber slides (Ibidi, USA) for 3 days prior to incubat with 20 μg/ml of anti-NS1 mAbs or irrelevant anti-E mAb (4G2) for 1 h on ice. Rabbit anti-human CD31 or anti-PECAM-1 (1:10 dilution, Santa Cruz Biotechnology, USA) was used as positive control of endothelial cell surface staining^11,13^. After washing three times with DMEM containing 2% FBS, cells were incubated for 30 min on ice with goat anti-mouse IgG or anti-rabbit IgG conjugated with Alexa Fluor 488 (1:500 dilution, Thermo Fisher Scientific) and Hoechst 33,342 (1:1000 dilution, Thermo Fisher Scientific) for staining of nuclei. Stained cells were visualized and imaged with a Carl Zeiss LSM800 with Airyscan confocal microscope. The fluorescence intensity of staining (Alexa Fluor 488) on the cell surface for each clone was determined using Zeiss microscopy Zen imaging software. The fluorescence intensity of the specific protein signal was normalized by nuclear staining intensity from the entire area of each captured image.

### Binding affinity of anti-NS1 antibodies to DENV1-4 NS1 proteins by Surface plasmon resonance technology

The affinity and kinetics of binding between NS1 proteins and anti-NS1 antibodies were analyzed using a Biacore X100 instrument (GE Healthcare) as previously described^29^. Briefly, recombinant NS1 protein (500 response unit; RU) of each DENV serotype was captured onto the surface of a Sensor Chip CM5 (GE Healthcare). Then, anti-NS1 mAbs (50 nM in running buffer of 1xHBS-N and 0.005% P20) were injected into the chip for 360 s and for 720 s. The sensorgrams and affinity constants (K_D_) were calculated by Biacore X100 software. Three replications were performed for each DENV serotype interaction.

## Supplementary Information

Supplementary Figure 1.

Supplementary Figure 2.

Supplementary Tables.

## Data Availability

All data generated or analyzed during this study are included in this article and its supplementary information files.
