# Supplementary material for: Differential critical residues on the overlapped region of the non-structural protein-1 recognized by flavivirus and dengue virus cross-reactive monoclonal antibodies
Source: Sci Rep. 2022 Dec 13;12:21548. doi: 10.1038/s41598-022-26097-y (PMC9747715; doi:10.1038/s41598-022-26097-y)
Supplement: Supplementary file 1 — Supplementary Figure 1. [file 41598_2022_26097_MOESM1_ESM.pdf]

Supplementary information

Original figures of Western blot assays

Original images for Fig 1b:

Western blot of anti-6His, 2E11 and 1F11 (at 100 sec. exposure time)

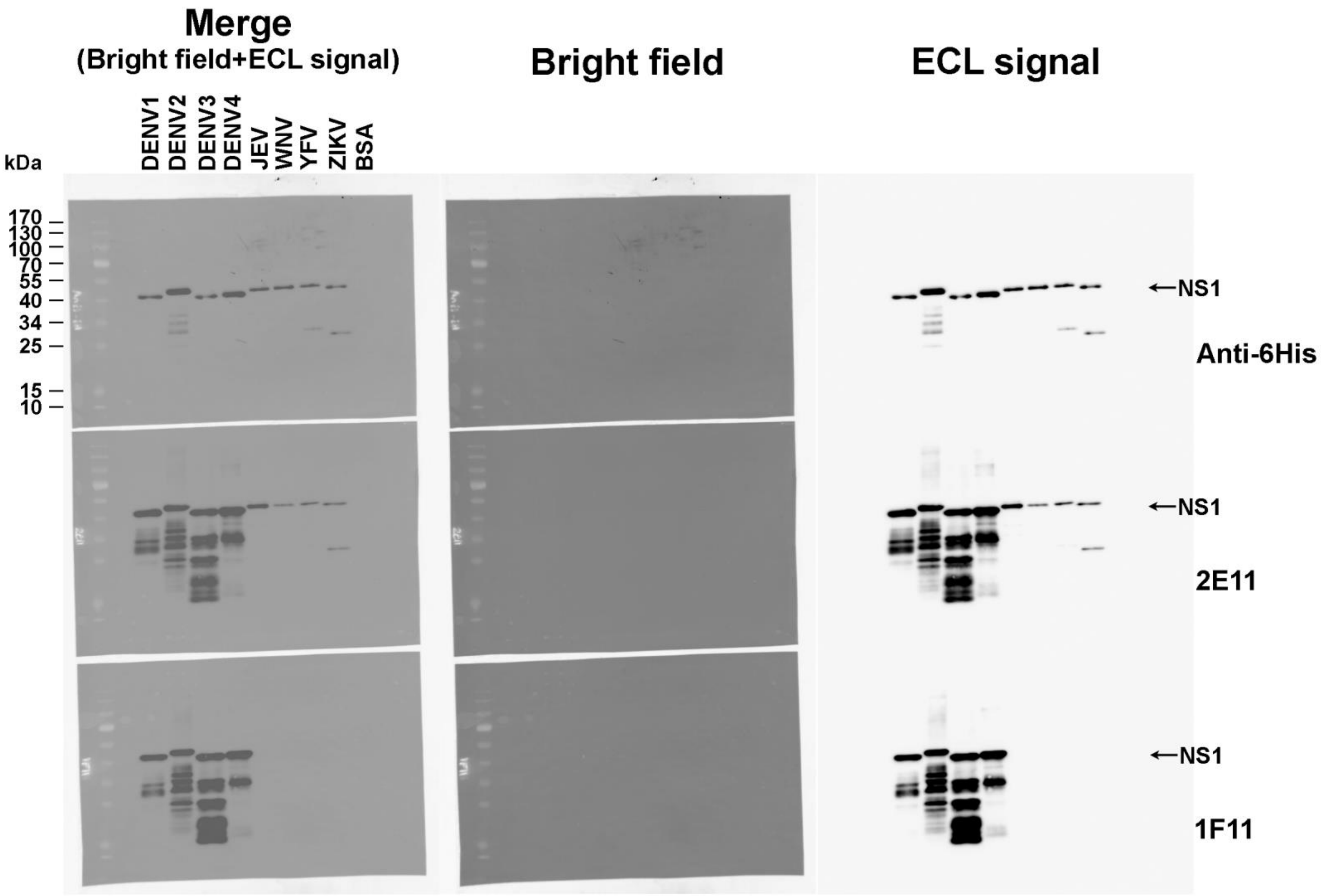

Original images for Fig 1b:

Western blot of 2E3, 1B2 and 4D2 (at 100 sec. exposure time)

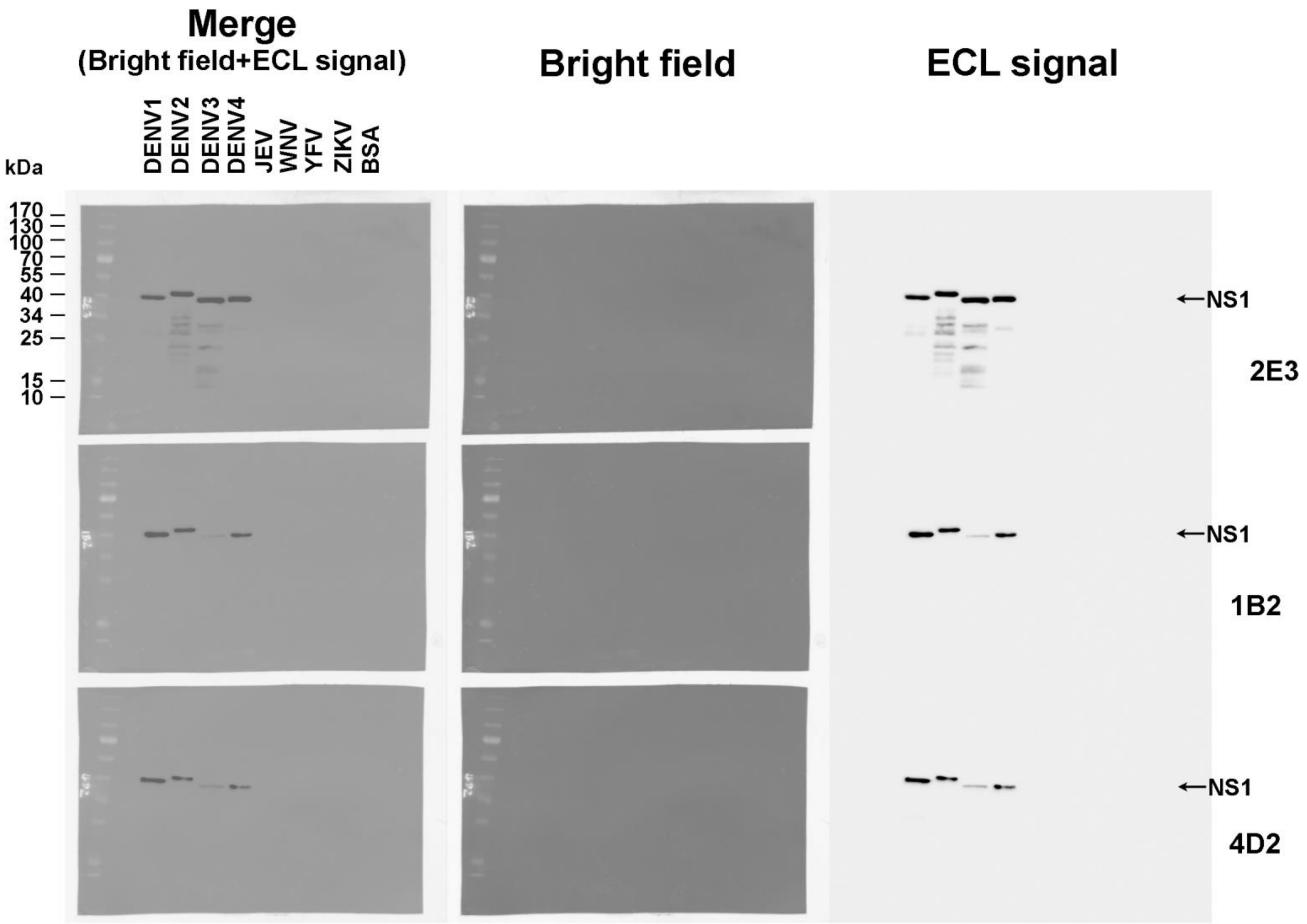

Original images for Fig 1b:

- Western blot of anti-6His and 5 mAbs (at 10, 50 and 100 sec. exposure times)

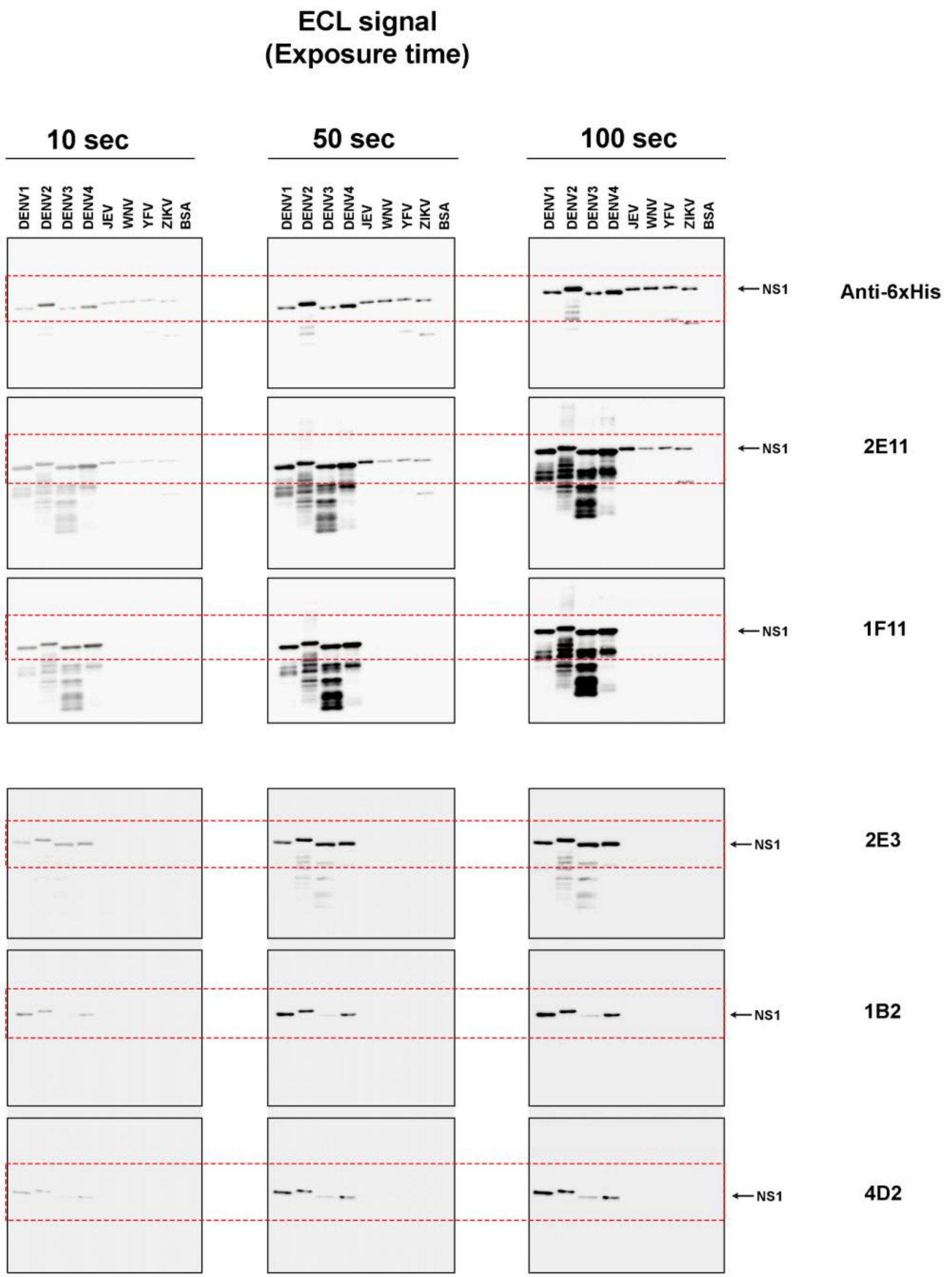

- SDS-PAGE with Coomassie blue staining

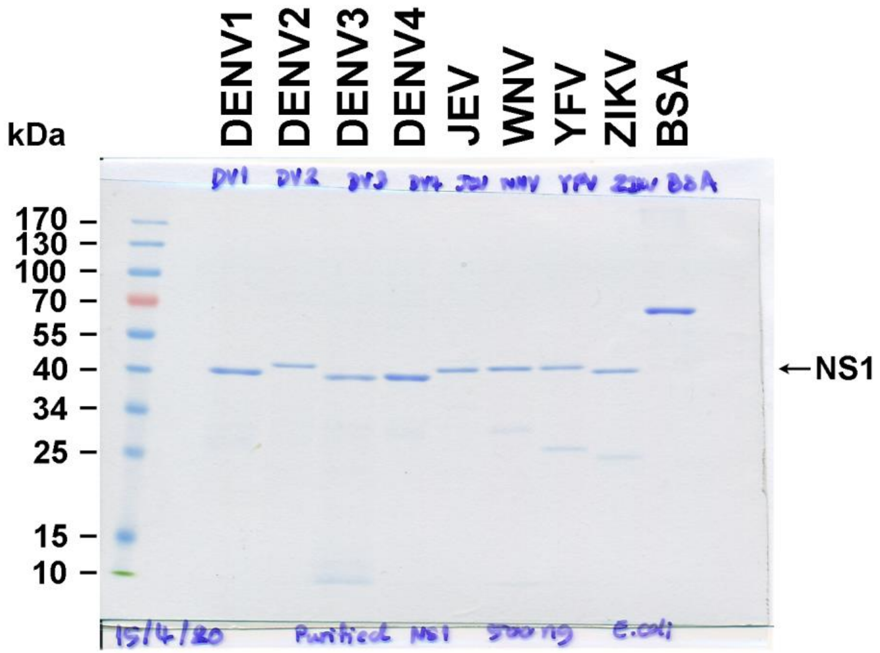

Original images for Fig 1c:

Western blot of 1F11

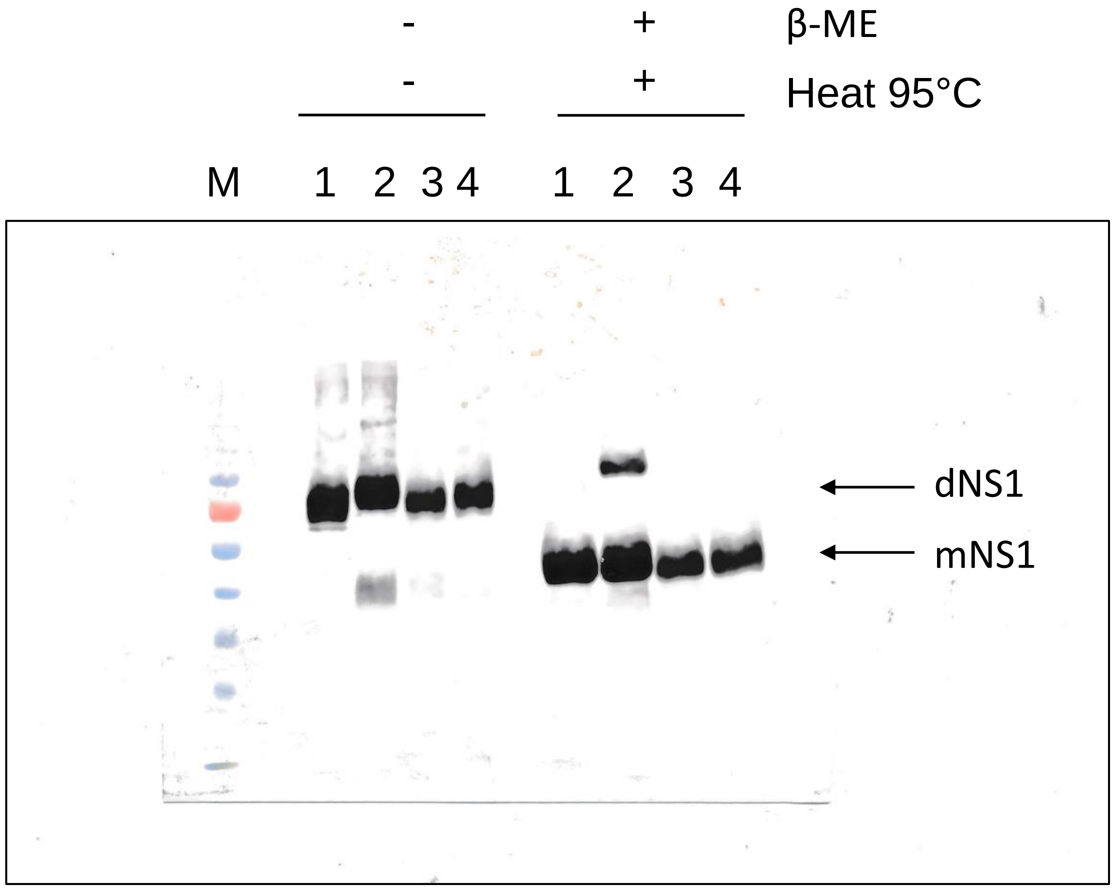

M = marker

1 = DENV1-NS1

2 = DENV2-NS1

3 = DENV3-NS1

4 = DENV4-NS1

Original images for Fig 2c:

Western blot of 2E11, 1F11, 2E3, 1B2, 4D2 and anti-6His

M 1 2 3 4 5 M 1 2 3 4 5 M 1 2 3 4 5 M 1 2 3 4 5 M 1 2 3 4 5 M 1 2 3 4 5

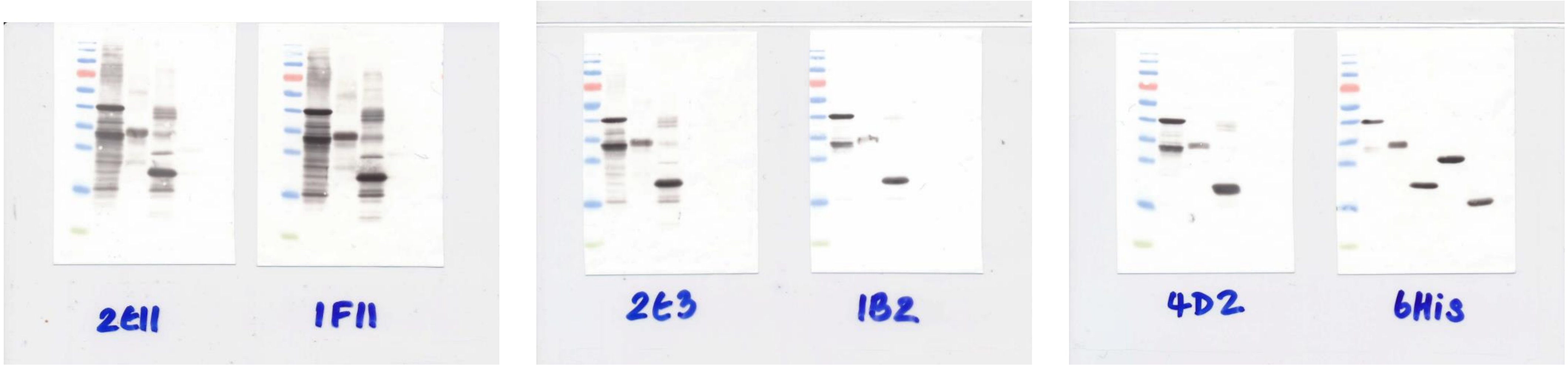

2E11 1F11 2E3 1B2 4D2 anti-6His

M = marker
1 = DENV2-NS1 full length
2 = NS1 FRI-II (aa1-273)
3 = NS1-FRI (aa1-157)
4 = NS1-FRII-III (aa158-352)
5 = NS1-FRIII (aa236-352)

Original images for Fig 4a:
Western blot of 2E11

M	Marker
1	Vector
2	D2NS1 wt
3	T109A
4	E110A
5	L111A
6	K112A
7	Y113A
8	S114A
9	W115A
10	K116A
11	T117A
12	W118A

M	Marker
13	G119A
14	K120A
15	A121G
16	K122A
17	M123A
18	L124A
19	S125A
20	T126A
21	E127A
22	S128A
23	H129A
24	N130A

2E11

Right

Left

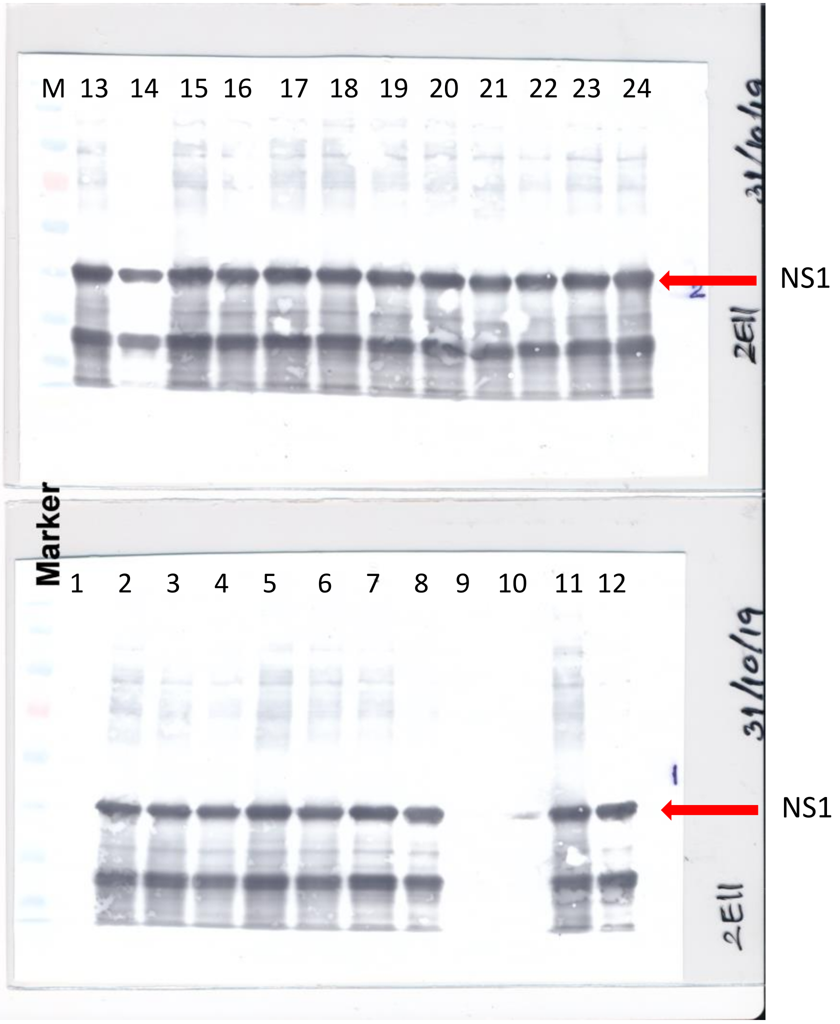

Original images for Fig 4a:

Western blot of 6-His and 1F11

M	Marker
1	Vector
2	D2NS1 wt
3	T109A
4	E110A
5	L111A
6	K112A
7	Y113A
8	S114A
9	W115A
10	K116A
11	T117A
12	W118A

M	Marker
13	G119A
14	K120A
15	A121G
16	K122A
17	M123A
18	L124A
19	S125A
20	T126A
21	E127A
22	S128A
23	H129A
24	N130A

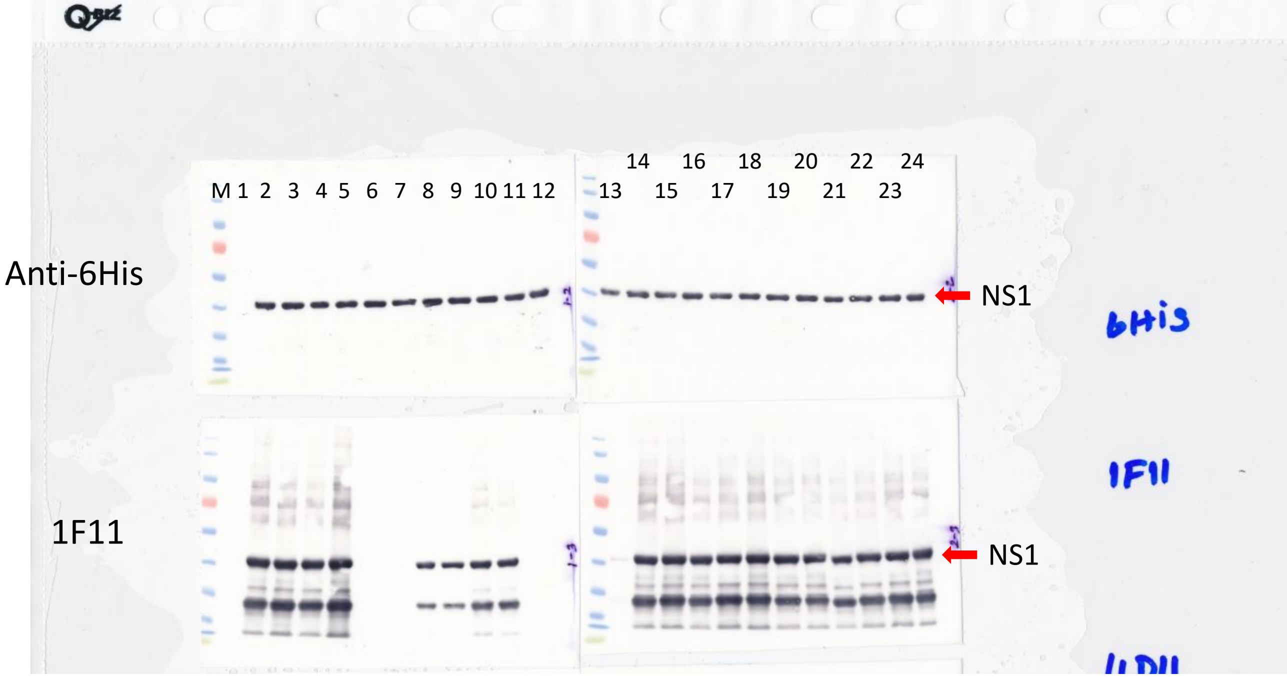

Original images for Fig 4a:

Western blot of 4D2, 1B2 and 2E3

M	Marker
1	Vector
2	D2NS1 wt
3	T109A
4	E110A
5	L111A
6	K112A
7	Y113A
8	S114A
9	W115A
10	K116A
11	T117A
12	W118A

M	Marker
13	G119A
14	K120A
15	A121G
16	K122A
17	M123A
18	L124A
19	S125A
20	T126A
21	E127A
22	S128A
23	H129A
24	N130A

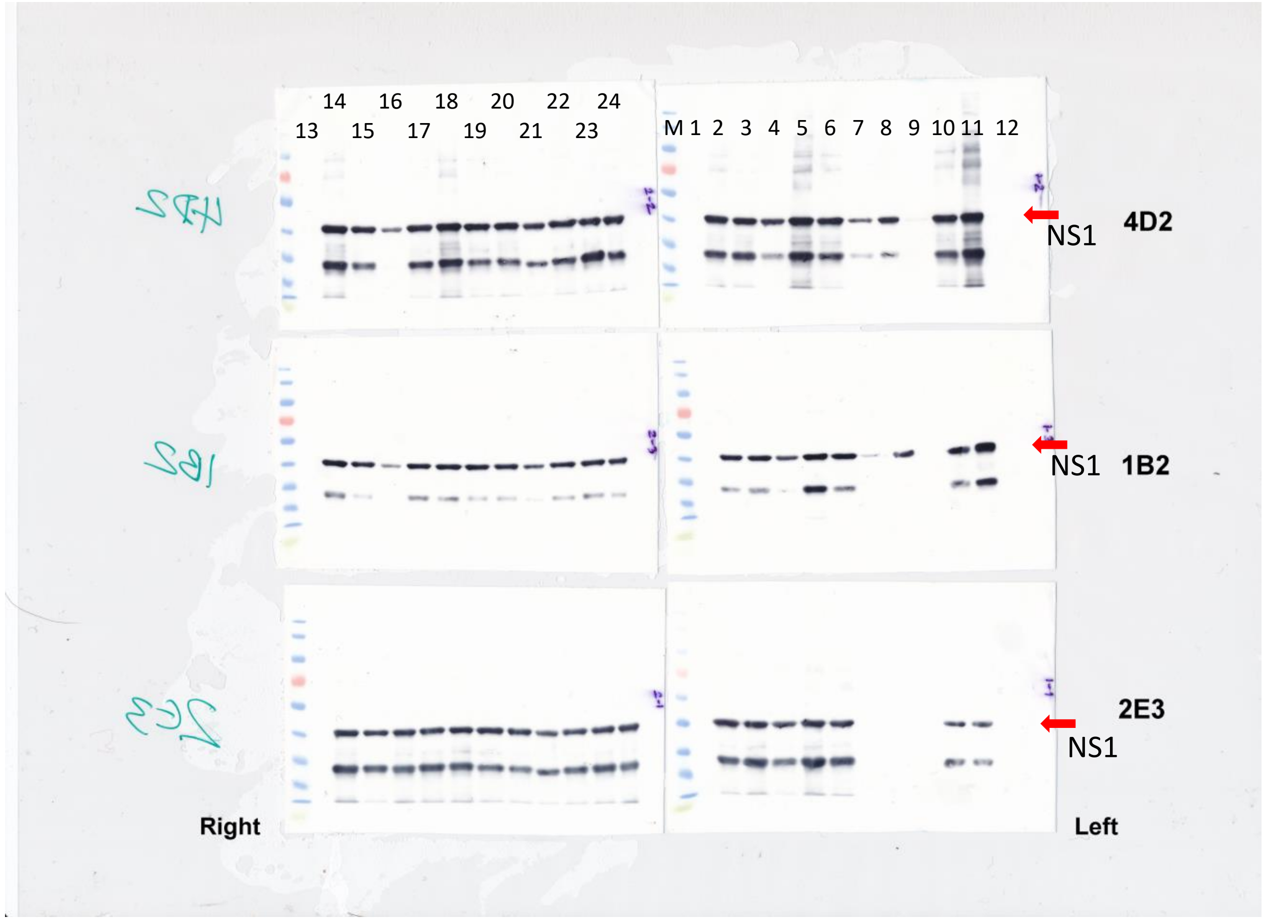

Original images for Fig 4a:

SDS-PAGE with Coomassie blue staining

M	Marker
1	Vector
2	D2NS1 wt
3	T109A
4	E110A
5	L111A
6	K112A
7	Y113A
8	S114A
9	W115A
10	K116A
11	T117A
12	W118A

M	Marker
13	G119A
14	K120A
15	A121G
16	K122A
17	M123A
18	L124A
19	S125A
20	T126A
21	E127A
22	S128A
23	H129A
24	N130A

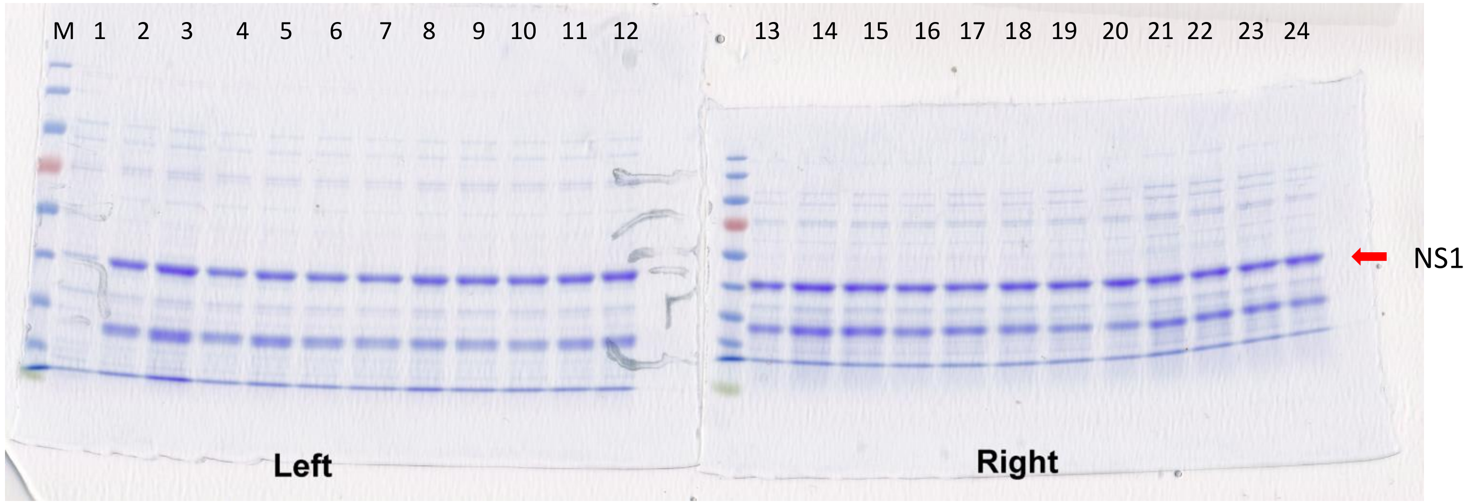

Original images for Fig 6b:

Western blot of anti-6His, 2E11, 1F11, 2E3, 1B2 and 4D2

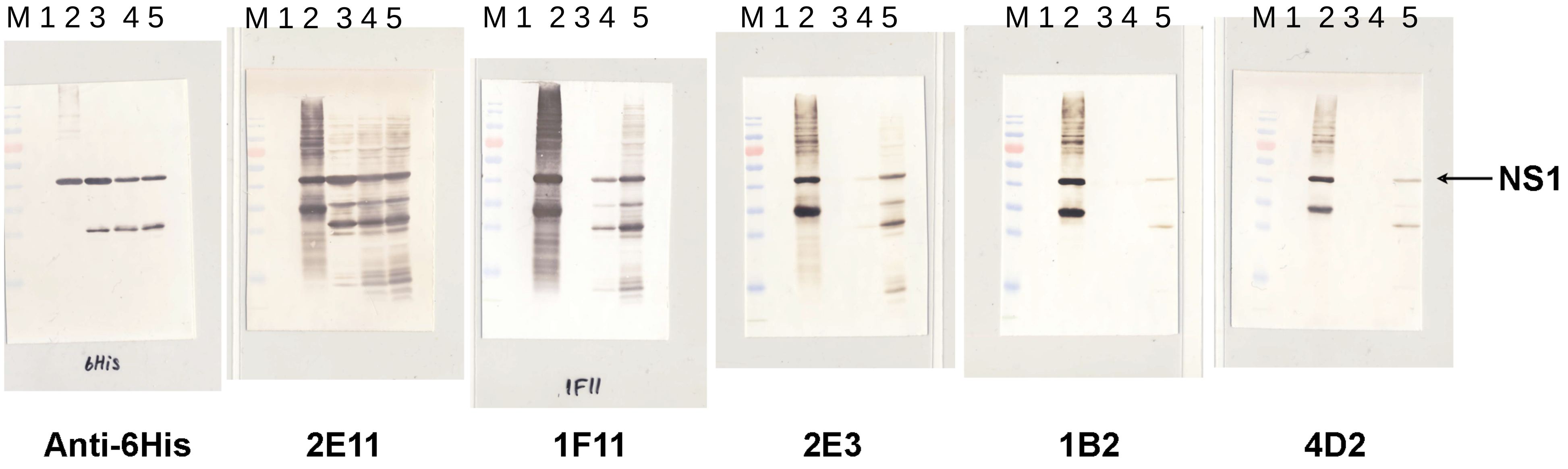

M = marker

1 = Mock E. coli lysate

2 = DENV2-NS1 wt

3 = ZIKV-NS1 wt

4 = ZIKV-NS1 KYS

5 = ZIKV-NS1 KYS/AK
