# Supplementary material for: Differential critical residues on the overlapped region of the non-structural protein-1 recognized by flavivirus and dengue virus cross-reactive monoclonal antibodies
Source: Sci Rep. 2022 Dec 13;12:21548. doi: 10.1038/s41598-022-26097-y (PMC9747715; doi:10.1038/s41598-022-26097-y)
Supplement: Supplementary file 2 — Supplementary Figure 2. [file 41598_2022_26097_MOESM2_ESM.pdf]

Supplementary Figures

Differential critical residues on the overlapped region of the non-structural protein-1 recognized by flavivirus and dengue virus cross-reactive monoclonal antibodies

Prasit Luangaram[1,2], Chamaiporn Tamdet[3], Chananya Saengwong[3], Tanapan Prommool[1,2], Romchat Kraivong[1,2,5], Napon Nilchan[1,2,5] Nuntaya Punyadee[2,4,5], Panisadee Avirutnan[2,4,5], Chatchawan Srisawat[6], Prida Malasit[2,4,5], Watchara Kasinrerk[7,8], Chunya Puttikhunt[1,2,5*]

[1]*Molecular Biology of Dengue and Flaviviruses Research Team, Medical Molecular Biotechnology Research Group, National Center for Genetic Engineering and Biotechnology (BIOTEC), National Science and Technology Development Agency (NSTDA), Pathum Thani, Thailand*

[2]*Medical Biotechnology Research Unit, BIOTEC, NSTDA, Bangkok, Thailand.*

[3]*Graduate Program, Department of Immunology, Faculty of Medicine, Siriraj Hospital, Mahidol University, Bangkok, Thailand.*

[4]*Division of Dengue Hemorrhagic Fever Research, Faculty of Medicine Siriraj Hospital, Mahidol University, Bangkok, Thailand*

[5]*Siriraj Center of Research Excellence in Dengue and Emerging Pathogens, Faculty of Medicine Siriraj Hospital, Mahidol University, Bangkok, Thailand*

[6]*Department of Biochemistry, Faculty of Medicine Siriraj Hospital, Mahidol University, Bangkok, Thailand*

[7]*Biomedical Technology Research Center, BIOTEC, NSTDA, Chiang Mai, Thailand*

[8]*Division of Clinical Immunology, Department of Medical Technology, Faculty of Associated Medical Sciences, Chiang Mai University, Chiang Mai, Thailand*

*Corresponding author **:**Chunya Puttikhunt

E-mail: chunyapk@biotec.or.th (C.P.)

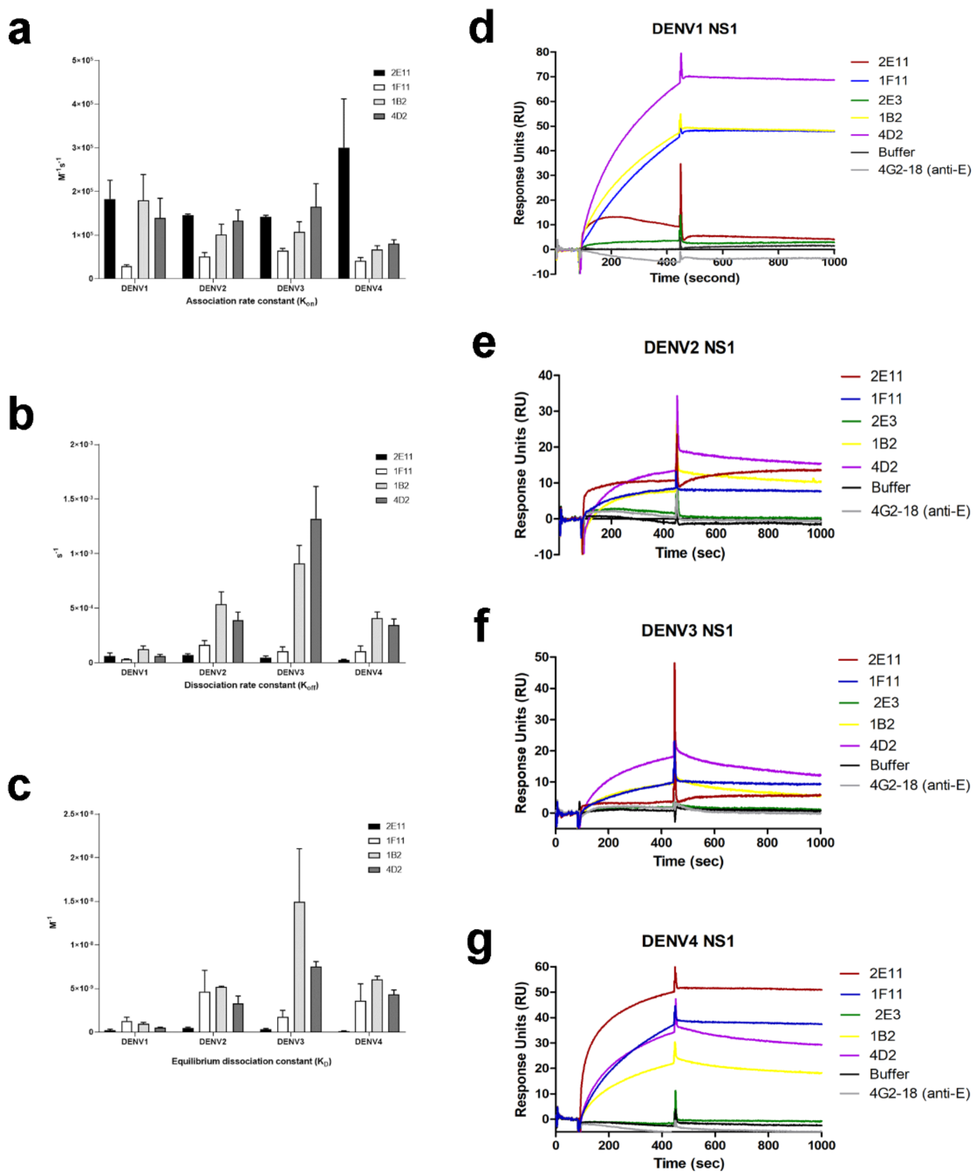

Supplementary Figure S1. Comparative antibody binding kinetics with DENV NS1 using surface plasmon resonance technology. Biacore X100 analyses of association rate constant (K_{on}) / dissociation rate constant (K_{off}) / equilibrium dissociation constant (K_D) of NS1-antibody binding (a-c). Sensogram of antibody to DENV1-4 NS1 over time course (d-g). Mouse 4G2-18 mAb (anti-E protein) and HBS-P buffer were included as negative controls for the calculation.

a

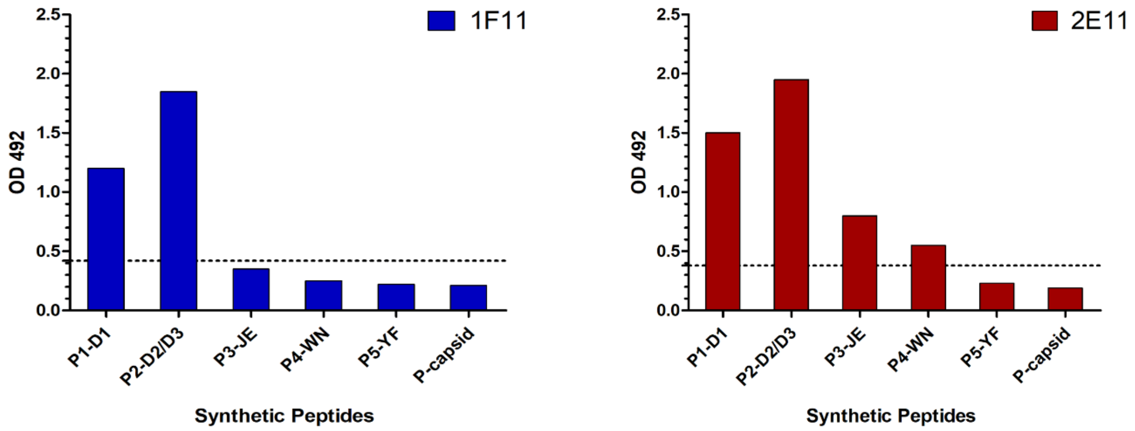

b

c

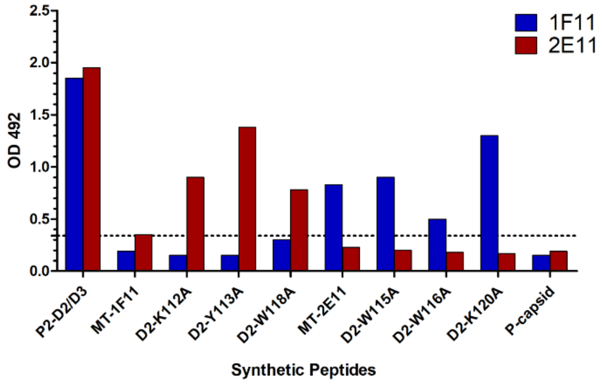

d

Peptide	Flaviviruses	Peptide sequences	NS1 residues
P1-D1	DENV1	EHKYSWKSWGKAK	110-112
P2-D2/3	DENV2 and 3	ELKYSWKTGWKAK	110-122
P3-JE	JEV	KFEMGWKAWGKSI	110-122
P4-WNV	WNV	KLEIGWKAWGKSI	110-122
P5-YFV	YFV	GLQYGWKTWGKNL	110-122
MT-1F11	DENV2	ELAASWKTAGKAK	110-122
D2-K112A	DENV2	ELAYSWKTWGKAK	110-122
D2-K113A	DENV2	ELKASWKTWGKAK	110-122
D2-W118A	DENV2	ELKYSWKTAGKAK	110-122
MT-2E11	DENV2	ELKYSAATWGAAK	110-122
D2-W115A	DENV2	ELKYSAKTWGKAK	110-122
D2-K116A	DENV2	ELKYSWATWGKAK	110-122
D2-K120A	DENV2	ELKYSWKTWGAAK	110-122
P-capsid	DENV2	NNQRKKAKNTPFNMLKRERN	C (1-20)

Supplementary Figure S2. Reactivity of anti-NS1 antibodies to synthetic oligopeptides derived from DENV2 NS1 and its mutants by ELISA. A set of 13-mer oligopeptides corresponding to aa 110−122 of NS1 from DENV2 (P2-D2/3) and other flaviviruses; DENV1 (P1-D1), JEV (P3-JE), WNV (P4-WN), YF (P5-YF) were reacted with 1F11 (a) or 2E11 (b). The other set of P2-D2/3 oligopeptide mutants of which critical binding residues for 1F11 (MT-1F11, D2-K112A, D2-Y113A, and D2-W118A) and 2E11 (MT-2E11, D2-W115A, D2-K116A, and D2-K120A) were changed to alanine were also reacted with 1F11 or 2E11 (c). ELISA was performed with a fixed concentration of coated synthetic peptides (10 μg/ml) and purified anti-NS1 antibodies (20 μg/ml). The peptide derived from aa 1-20 of DENV2 capsid protein (P-Capsid) was included as a negative control. Dashed line indicates the cut-off value as obtained by twice OD reading to P-capsid. (d) The detailed amino acid sequences of tested oligopeptides.

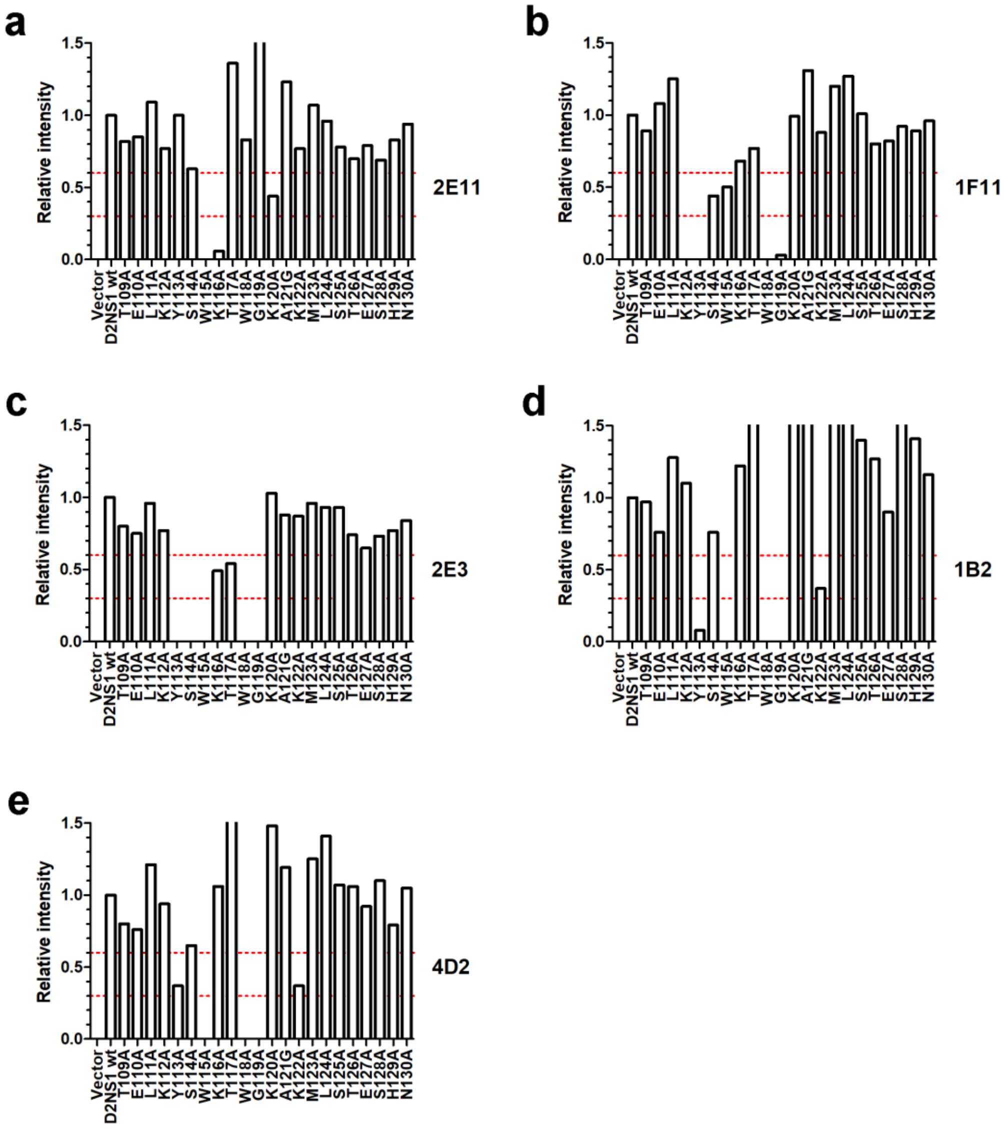

Supplementary Figure S3. Analysis of alanine-substituted NS1 mutants by western blot analysis. Band intensity of NS1 mutants (aa 109-130) as shown in Fig 6A were measured and normalized to that of NS1 wild type. Relative intensity values (mutant: wild-type) were plotted for all mutants tested with each anti-NS1 mAb. (A) 2E11, (B) 1F11, (C) 2E3, (D) 1B2, (E) 4D2. Relative intensity at 0.3 and 0.6 (red dotted lines) were used to classify the effect of the mutations as strong (below 0.3), moderate (between 0.3-0.6) or negligible (over 0.6).

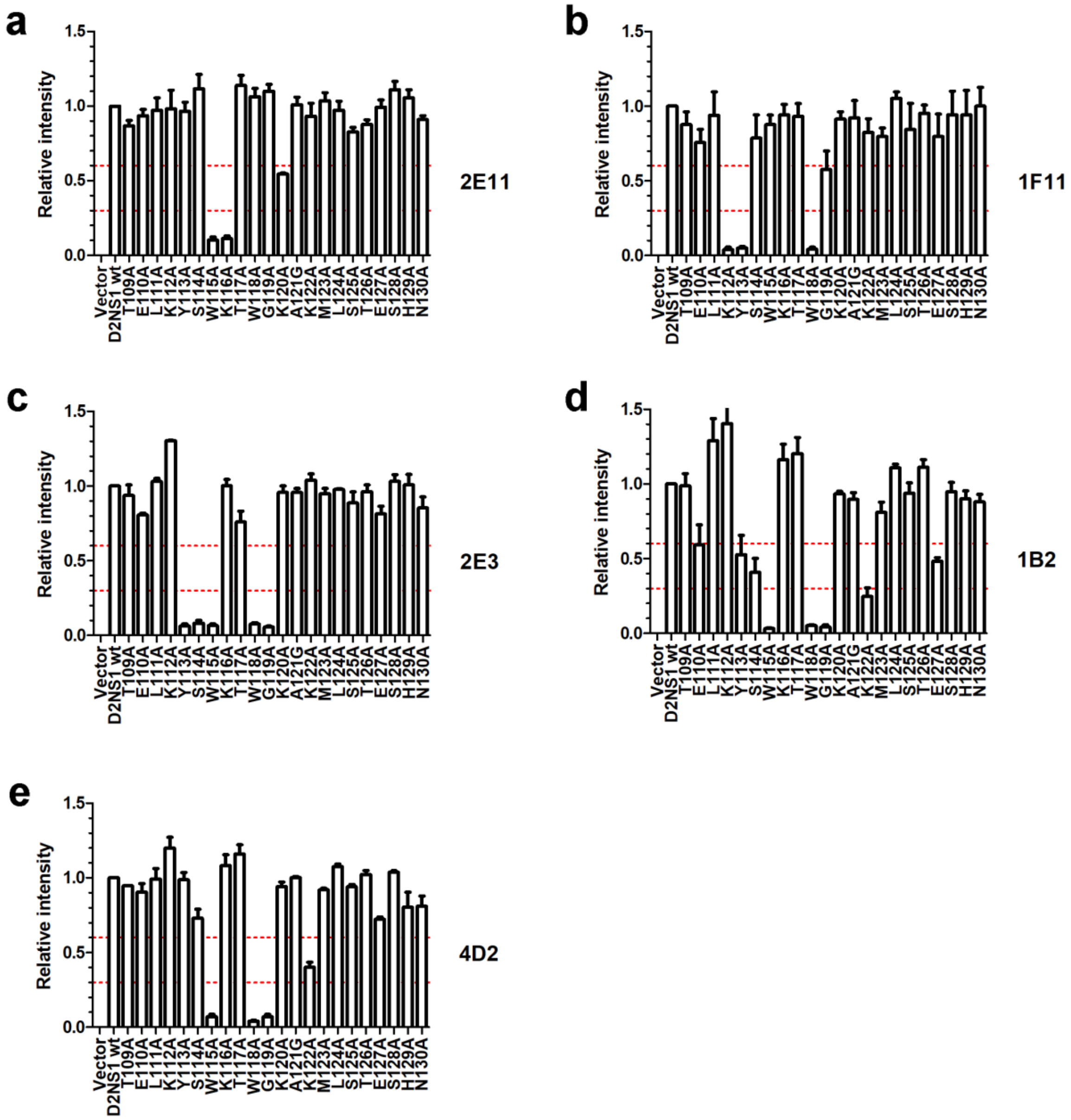

Supplementary Figure S4. **Analysis of alanine-substituted NS1 mutant by dot blot analysis.** Dot intensity of NS1 mutants (aa 109-130) as shown in Fig 6B were measured and normalized to that of NS1 wild type. Relative intensity values (mutant: wild-type) were plotted for all mutants tested with each anti-NS1 mAb. (A) 2E11, (B) 1F11, (C) 2E3, (D) 1B2, (E) 4D2. Relative intensity at 0.3 and 0.6 (red dotted lines) were used to classify the effect of the mutations as strong (below 0.3), moderate (between 0.3-0.6) or negligible (over 0.6).

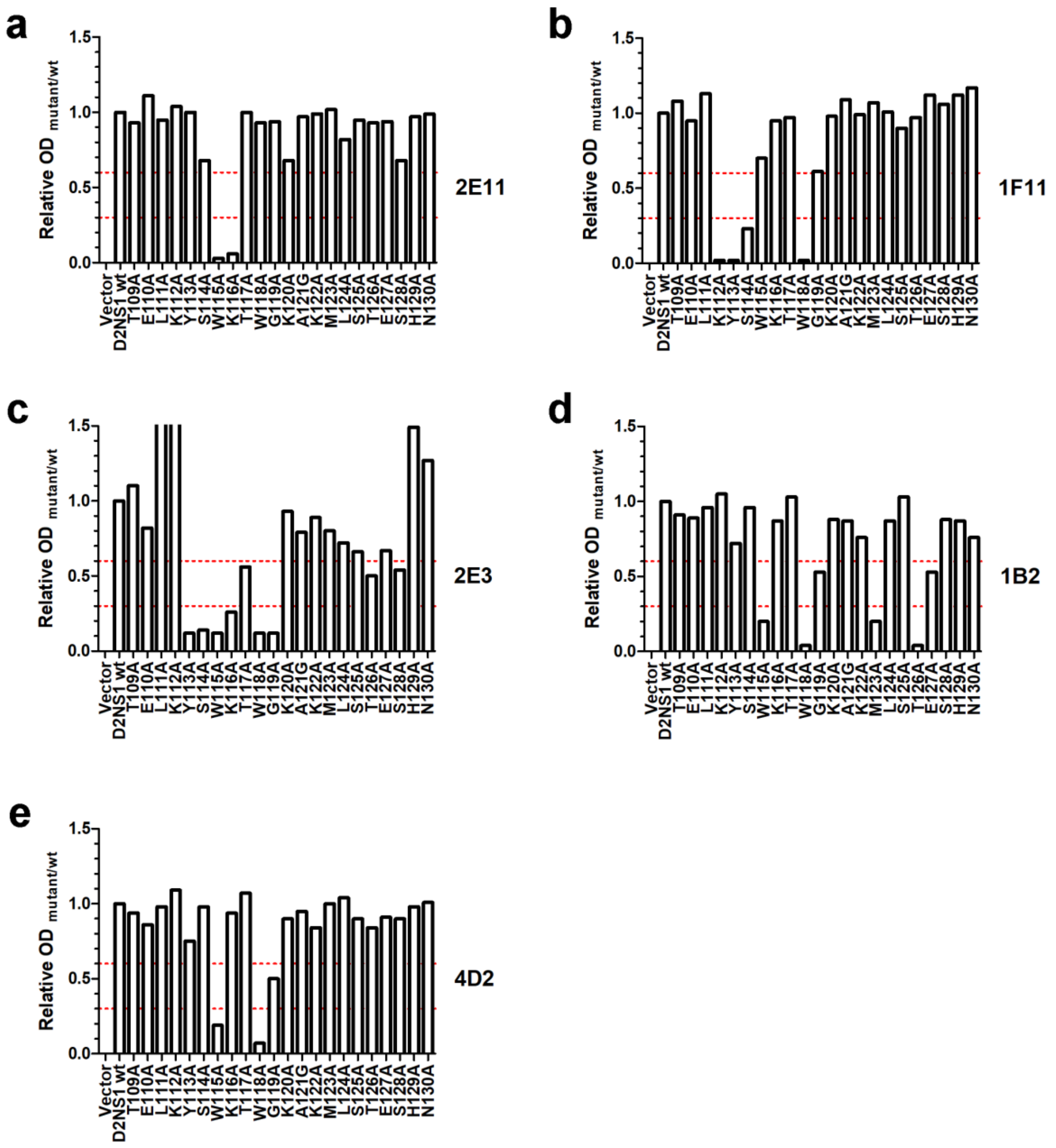

Supplementary Figure S5. **Analysis of alanine-substituted NS1 mutant by ELISA.** The OD reading of NS1 mutants (aa 109-130) as shown in Fig 6C were measured and normalized to that of NS1 wild type. Relative OD values (mutant: wild-type) were plotted for all mutants tested with each anti-NS1 mAb. (A) 2E11, (B) 1F11, (C) 2E3, (D) 1B2, (E) 4D2. Relative OD at 0.3 and 0.6 (red dotted lines) were used to classify the effect of the mutations as strong (below 0.3), moderate (between 0.3-0.6) or negligible (over 0.6).

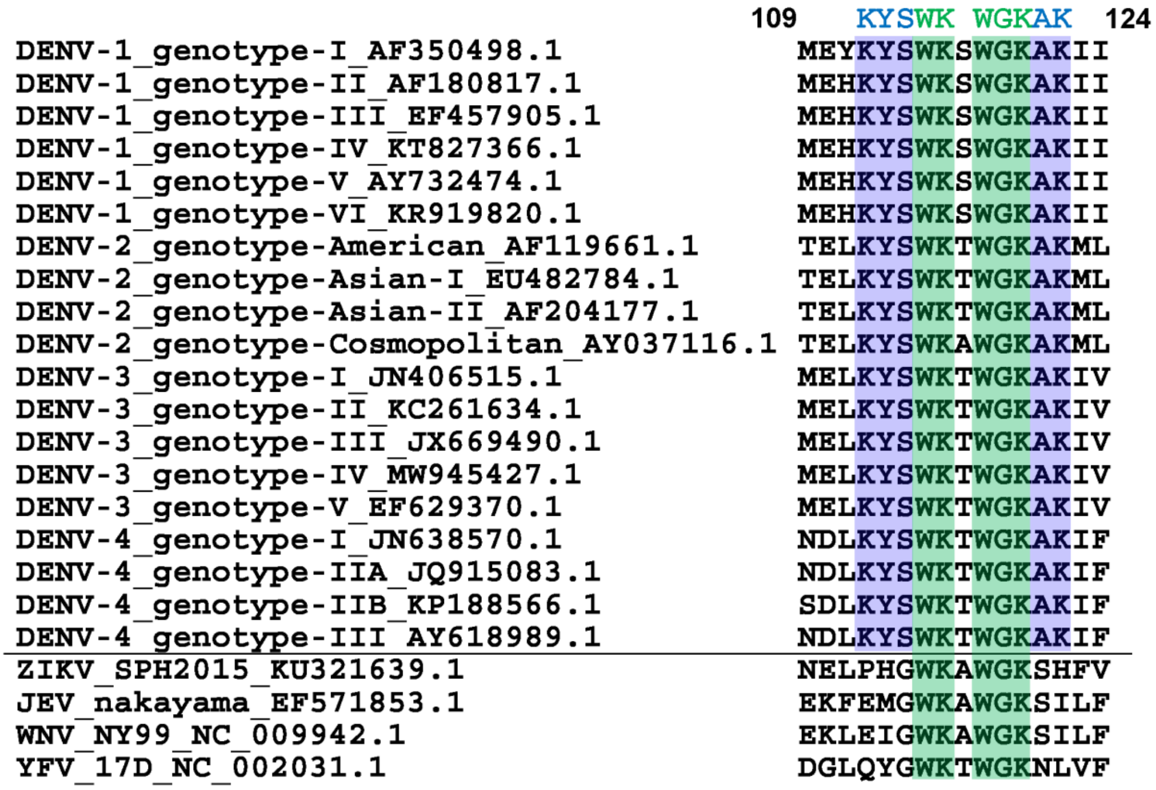

Supplementary Figure S6. Amino acid alignments of flaviviruses NS1 residues 109-124. DENV strains belonged to different genotypes of DENV 1-4 as well as other flaviviruses (ZIKV, JEV, WNV, YFV) and their corresponding NS1 residues 109-124 were aligned. DENV conserved residues KYS/AK (blue) and flavivirus conserved residues WK-WGK (green) are highlighted.

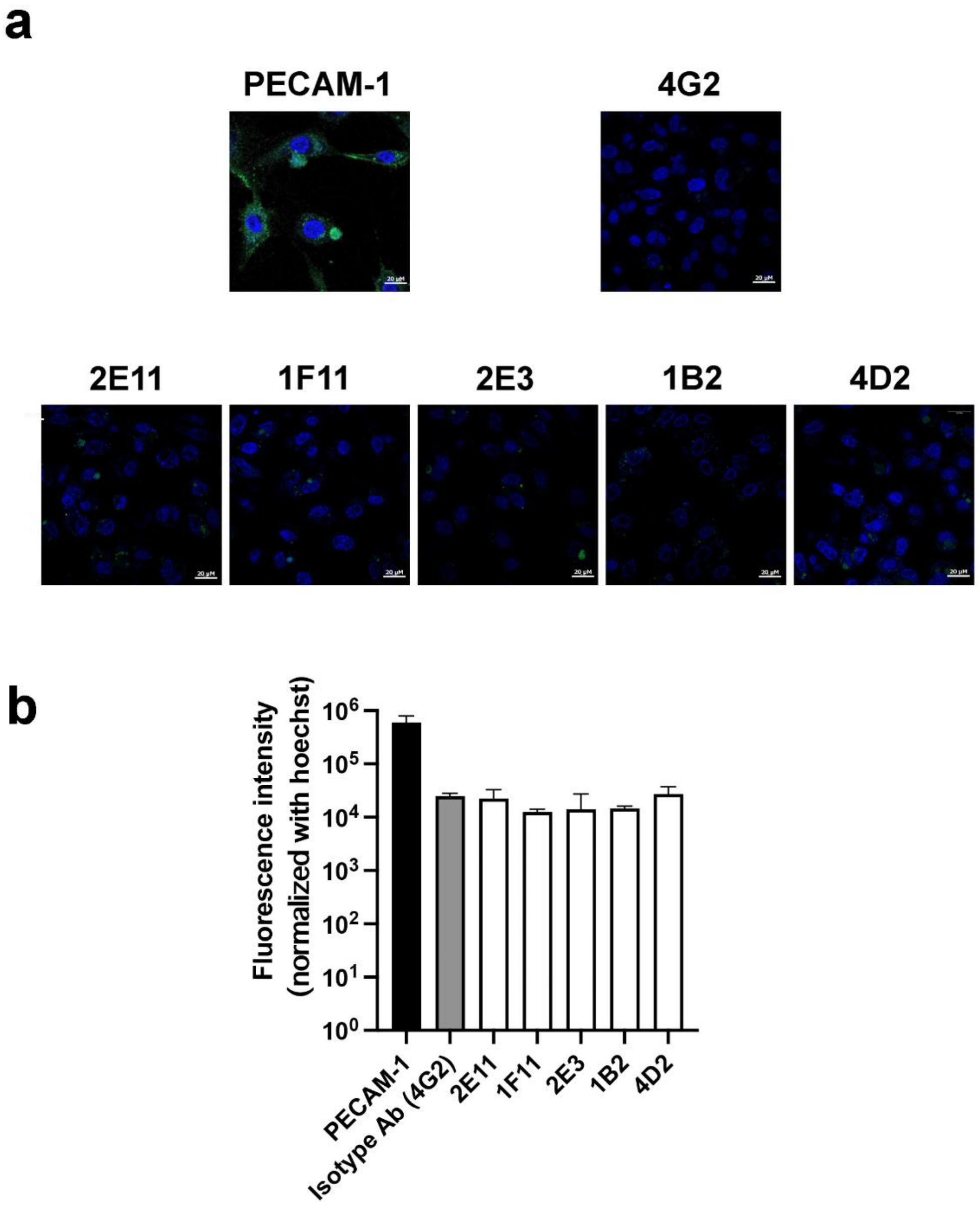

Supplementary Figure S7. Indirect immunofluorescent staining of anti-NS1 antibodies to LYRIC protein on endothelial cell surfaces. Human umbilical vein endothelial cells (HUVECs) were fixed, and surface stained by anti-NS1 antibodies, followed with anti- IgG conjugated with Alexa Fluor 488 (dilution 1:500). PECAM-1 is a control antibody recognized the host LYRIC epitope. 4G2 is an irrelevant control antibody recognized DENV E protein. Cell nuclei was stained by Hoechst 33342 (dilution 1:1000). (A) The images of cell surface (green) and nuclear staining (blue) as visualized by confocal microscopy. Scale bars are indicated on the bottom right of each panel. (B) The log10-transformed fluorescence intensity of each antibody to the cell surface comparing to PECAM-1 (positive control, black bar) and 4G2 (negative control, gray bar).
